# The Associations of Selenoprotein Genetic Variants with the Risks of Colorectal Adenoma and Colorectal Cancer: Case–Control Studies in Irish and Czech Populations

**DOI:** 10.3390/nu14132718

**Published:** 2022-06-29

**Authors:** Maryam Mukhtar, Niall Ashfield, Ludmila Vodickova, Veronika Vymetalkova, Miroslav Levy, Václav Liska, Jan Bruha, Petra Bendova, Jacintha O’Sullivan, Glen Doherty, Kieran Sheahan, Blathnaid Nolan, Pavel Vodicka, David J. Hughes

**Affiliations:** 1 Cancer Biology and Therapeutics Group, School of Biomolecular and Biomedical Science, UCD Conway Institute, University College Dublin, D04 V1W8 Dublin, Ireland; m.mukhtar29@gmail.com (M.M.); niall.ashfield@gmail.com (N.A.); 2 Institute of Biology and Medical Genetics, First Faculty of Medicine, Charles University, 121 08 Prague, Czech Republic; ludmila.vodickova@iem.cas.cz (L.V.); vpolakova@biomed.cas.cz (V.V.); pavel.vodicka@iem.cas.cz (P.V.); 3 Biomedical Centre, Faculty of Medicine in Pilsen, Charles University, 323 00 Pilsen, Czech Republic; vena.liska@skaut.cz (V.L.); bruhaj@fnplzen.cz (J.B.); 4 Institute of Experimental Medicine ASCR, 142 20 Prague, Czech Republic; petra.bendova@email.cz; 5 Department of Surgery, First Faculty of Medicine, Charles University and Thomayer Hospital, 121 08 Prague, Czech Republic; miroslav.levy@ftn.cz; 6 Trinity Translational Medicine Institute, Department of Surgery, Trinity College Dublin and St. James’s Hospital, D08 NHY1 Dublin, Ireland; osullij4@tcd.ie; 7 Centre for Colorectal Disease, St. Vincent’s University Hospital, D04 T6F4 Dublin, Ireland; glen.doherty@ucd.ie (G.D.); ksheahan@svhg.ie (K.S.); b.nolan2@st-vincents.ie (B.N.)

**Keywords:** Selenium, selenoprotein gene variation, Selenium pathway, colorectal neoplasms, case–control cohorts, colorectal cancer risk

## Abstract

Background: Selenium manifests its biological effects through its incorporation into selenoproteins, which play several roles in countering oxidative and inflammatory responses implicated in colorectal carcinogenesis. Selenoprotein genetic variants may contribute to colorectal cancer (CRC) development, as we previously observed for SNP variants in a large European prospective study and a Czech case–control cohort. Methods: We tested if significantly associated selenoprotein gene SNPs from these studies were also associated with CRC risk in case–control studies from Ireland (colorectal neoplasia, i.e., cancer and adenoma cases: 450, controls: 461) and the Czech Republic (CRC cases: 718, controls: 646). Genotyping of 23 SNPs (20 in the Irish and 13 in the Czechs) was performed by competitive specific allele-specific PCR (KASPar). Multivariable adjusted logistic regression was used to assess the associations with CRC development. Results: We found significant associations with an increased CRC risk for rs5859 (*SELENOF*) and rs2972994 (*SELENOP*) in the Irish cohort but only with rs4802034 (*SELENOV*) in the Czechs. Significant associations were observed for rs5859 (*SELENOF*), rs4659382 (*SELENON*), rs2972994 (*SELENOP*), rs34713741 (*SELENOS*), and the related Se metabolism gene variant rs2275129 (*SEPHS1*) with advanced colorectal neoplasia development. However, none of these findings retained significance after multiple testing corrections. Conclusions: Several SNPs previously associated with CRC risk were also associated with CRC or colorectal neoplasia development in either the Irish or Czech cohorts. Selenoprotein gene variation may modify CRC risk across diverse European populations, although the specific variants may differ.

## 1. Introduction

Among tumors across Europe, colorectal cancer (CRC) is the second leading type of cancer for both incidence and mortality rates [1]. These rates in Ireland and also the Czech Republic are higher than the EU average [1]. To markedly reverse these rates, it is vital to improve prevention and early detection, as most CRCs are still diagnosed at advanced stages.

The marked geographic variation worldwide in the dietary availability of the essential micronutrient selenium (Se) results in a lower Se status for many populations, including those across Europe, compared with much of North America [2,3]. The suboptimal intake of Se has been associated with an increased risk of several major diseases [2,3,4], including colorectal neoplasia (polyps and tumors) [5,6].

Se exerts its physiological functions and potential anti-carcinogenic properties through its incorporation into selenoproteins as the amino acid selenocysteine. The human selenoproteome is encoded by 25 separate genes that play key roles in cell protection from oxidative stress, redox control, and inflammatory responses [7,8]. The level of Se intake in Europe and other parts of the world is not adequate for the full expression of these protective selenoproteins [3,9], and this is thought to be the major mechanism through which Se can influence CRC development risk [5].

In all, around 50% of selenoprotein genes in populations from Asia, North America, and Europe harbor single nucleotide polymorphisms (SNPs) proposed to be associated with colorectal adenoma (CRA) development and CRC risk and recurrence risk, as well as impacting survival outcomes [10,11,12,13,14,15,16]. Among these, Méplan and colleagues (2010) first reported that genetic variants known to affect the functional activity of selenoproteins in the *GPX4*, *SELENOP*, and *SELENOS* genes were associated with CRC risk in a case–control study from the Czech Republic [11], a European country of known low Se status [17]. The largest study assessing common variation in 154 Se-related genes was conducted by Fedirko and colleagues [10] in a multi-center, European cohort study of 1420 cases and 1420 controls. Here, it was reported that several SNPs in selenoprotein and Se metabolic pathway genes may affect CRC development risk [10], alone or in combination with suboptimal Se status, ascertained by levels of Se and the selenoprotein P (SELENOP) Se transport protein. However, other studies in generally replete Se status populations have reported more equivocal findings. A prospective study from the Women’s Health Initiative (WHI) in the USA reported no major association with CRC risk for SNPs in five selenoproteins (*GPX1-4* and *SELENOP*) [18]. However, selenoprotein variants (in several pathway genes) were associated with CRC risk and survival outcomes in a large study of a USA population with generally adequate dietary Se intakes [16]. Thus, although no individual selenoprotein SNP variant (to our knowledge) has been confirmed in CRC–GWAS (reviewed by [14,15]), this probably reflects the complex interaction between Se intake and the genetic and environmental factors implicated in the etiology of CRC [19].

In this replication study, we analyzed the associations with CRA and CRC risk of several SNPs in selenoprotein genes (including known functional variants and other tagging SNPs) previously reported to be associated with CRC in European populations in studies led by two of the current study’s coauthors [10,11]. This study was conducted in two studies from the small and genetically homogeneous Irish (colorectal neoplasia cases: 450, controls: 461) and Czech (CRC cases: 718, controls: 646) populations, with the latter partially overlapping with a previous report [11].

## 2. Materials and Methods

### 2.1. Subjects and Case–Control Designations

This study examined two colorectal neoplasia study cohorts, from the Czech Republic and from Ireland. Selected baseline characteristics of both study populations are summarized in Table 1. The Czech cohort comprised 718 CRC cases and 646 controls collected at several oncology departments in the Czech Republic between September 2003 and December 2016. Controls were those requiring colonoscopy for various gastrointestinal complaints but who showed no evidence of malignant or idiopathic bowel disease and had no history of chronic illness requiring repeated hospitalization [20]. The Irish cohort comprised 450 cases of 251 CRC and 209 advanced adenomas (i.e., adenomas with high-grade dysplasia (HGD), adenomas with at least 20% tubular villous or villous features, all adenomas greater than 10 mm, and the presence of three or more adenomas) [21,22]. The control group consisted of blood donor controls (>50 years at blood collection) and subjects with a positive immunochemical fecal occult blood test (FIT) upon CRC screening but either with no detectable pathology after colonoscopy (*n* = 461) or with small (1–2 mm) hyperplastic polyps (*n* = 110). Cases and FIT screening samples were collected from Tallaght University Hospital from 2008 to 2011 from study volunteers within the departments of Gastroenterology and Surgery, and most were recruited patients with a positive FIT test from a CRC screening pilot program [23], in addition to further consecutive CRC patients from the surgery department. Blood samples (for DNA extraction) from an additional 198 incident CRC cases came from the CRC biobank of the UCD Centre for Colorectal Disease, St Vincent University Hospital, Dublin.

Colorectal neoplasia histology was verified by experienced pathologists at each study center. The clinical characteristics of the study cohorts, including age, sex, tumor localization and histology, and pathological tumor–node–metastasis (pTNM) stage (tumor stage, regional lymph node involvement, and distant metastasis) were taken from patients’ medical records. There were limited epidemiological data for the Irish study (restricted to age, sex, and family history). However, all Czech cohort study participants completed demographic/lifestyle questionnaires to provide information regarding their education, lifestyle habits, body mass index, diabetes, family/personal history of cancer, and long-term (at least six consecutive months) drug use. The data for these Czech samples were retrieved and acquired as previously described [24].

Cancer cases were coded using the 10th Revision of the International Classification of Diseases (ICD-10) and the second revision of the International Classification of Disease for Oncology (ICDO-2). Tumors that were overlapping or unspecified (C18.8 and C18.9) were not included in the assignment of proximal and distal colon tumors. Tumors for the proximal and distal colon as well as overlapping or unspecified tumors were combined to define tumors from the whole colon. Cancers of the rectum were defined as tumors occurring at the recto-sigmoid junction (C19) or the rectum (C20). Subjects with anal canal tumors (C21) were excluded from this study.

All patients provided informed consent according to the Helsinki Declaration (2013 update). The study received approval from the Ethical Committees of the St. James’s Hospital and Federated Dublin Voluntary Hospitals (Dublin, Ireland), and St Vincent’s Hospital Healthcare Group (Dublin, Ireland) and from the Ethical Committee of the Medical Faculty and Teaching Hospital in Pilsen, Czech Republic. All biosamples and patient data were coded to protect anonymity; blood samples and the extracted DNA were stored at −80 °C.

### 2.2. Blood Collection and DNA Extraction

For the Czech cohort, germline DNA was isolated from peripheral leucocytes within 4 weeks of blood sample collection using the standard proteinase K digestion, phenol/chloroform extraction, and ethanol precipitation and stored at −80 °C. Concentrations were then determined using a Nanodrop 2000c Spectrophotometer. Blood samples were taken from the Irish patients from Tallaght University Hospital 1 day before surgery/colonoscopy in EDTA-coated 6 mL VACUTAINER^®^ tubes, (Cruinn Diagnostics, Dublin, Ireland). The buffy coat layer was separated by centrifuging the samples at 2000× *g* for 10 min (within 4 h of collection), aliquoted into cryovials, and stored at −80 °C. For the CRC samples from St Vincent’s, the DNA was extracted from peripheral blood mononuclear cells that had been stored frozen at −80 °C. All DNA was extracted using the automated Autopure Instrumentation (Qiagen, Hilden, Germany) at St James University Hospital. A NanoDrop 2000c Spectrophotometer (ThermoScientific, Asheville, NC, USA) was used to quantify the extracted DNA.

### 2.3. Single Nucleotide Polymorphism Genotyping

Participants were genotyped for a total of 23 SNPs in 17 genes, of which 17 were in 13 selenoprotein genes, while the other 6 were in 6 wider Se-related pathway genes (antioxidant and redox reactions, apoptosis, and Wnt signaling). Ten of these (rs2074451 in *GPX4*, rs445870 in *GPX5*, rs11705137 in *SELENOM*, rs11247710 and rs4659382 in *SELENON*, rs2275129 in *SEPHS1*, rs11111979 in *TXNRD1*, rs4645887 in *BAX*, rs3813498 in *FOXO3,* and rs17265803 in *FRZB*) were genotyped in both the Irish and the Czech cohorts. Following previously published data in Czech populations [11], these 10 SNPs were genotyped only in the Irish samples (rs1050450 in *GPX1,* rs8177447 in *GPX3*, rs713041 in *GPX4*, rs5859 in *SELENOF*, rs2972994 and rs7579 in *SELENOP*, rs8177426 and rs34713741 in *SELENOS*, rs9605031 in *TXNRD2*, and rs4880 in *SOD2*). Finally, a further 3 SNPs (rs9818758 in *GPX1*, rs4802034 in *SELENOV,* and rs7953266 in *TXNRD1*) were genotyped only in the Czechs, as this CRC case–control cohort was primarily used to validate the results from our 2019 study in the European Prospective Investigation of Cancer and Nutrition (EPIC) [10]. Table S1 lists the allele frequencies for these SNPs observed in both the Czech and Irish study populations. The genotyping was carried out using a robust, competitive allele-specific PCR system (KASPar) by LGC Genomics (Hoddesdon, Hertfordshire, EN11 OEX, UK). Genotyping analysis was blinded to case–control status, and a random 2% of samples as duplicate quality controls were validated to ensure complete concordance. Samples with inconclusive or failed genotype calls were excluded from the analysis; the genotyping success rate was 94.3%. The genotype correlation between the duplicate samples was 100%. Details of allele probe sequences are available upon request.

For cases and controls combined, all SNP genotypes were in Hardy–Weinberg equilibrium (HWE) in both populations, except for *GPX4* rs713041, *TXNRD1* rs11111979, and *FRZB* rs17265803 in the Irish cohort and *SELENOV* rs4802034 in the Czech cohort (Table S1). When separated by case–control status, all studied SNPs were in HWE in the Czech controls, while *SELENOV* rs4802034 was not in HWE in the Czech cases. Finally, rs713041 and rs11111979 were not in HWE in the Irish cases (with polyps either included or excluded), and *SELENOS* rs17265803 was not in HWE in the cases with polyps excluded.

### 2.4. Statistical Analyses

To perform the case–control comparisons in the Irish colorectal neoplasia cohort, we used two different case–control allocation models dependent on the inclusion of small hyperplastic polyps (<2 mm) in the control or pathology (case) group. This is because most of these small hyperplastic polyps have generally been regarded as benign and are found in 20–40% of people over the age of 50 [25], but there is some evidence to suggest that these polyps may be precancerous [26].

The association between individual SNPs and CRC risk was estimated by unconditional logistic regression to calculate the odds ratio (OR) and 95% confidence interval (CI), adjusting for age (as a continuous variable) and sex. Associations in the Czech cohort were also adjusted for the individual’s smoking habits, body mass index (BMI), and alcohol consumption. ORs are presented using the most frequent homozygous genotype as a reference. Three standard genetic analysis models were tested for disease penetrance: common dominant, common recessive, and additive models [27]. Subgroup analyses by sex and anatomical subsite of the colorectum (colon and rectum) were conducted. A two-sided *p*-value below 0.05 was considered statistically significant. Multiple testing corrections considering the false discovery rate (FDR) were performed using the Benjamin–Hochberg procedure [28]. Analyses were conducted using the R 4.1.2 and R packages (R Foundation for Statistical Computing, Vienna, Austria; http://www.R-project.org/ (accessed on 1 November 2021)) and PLINK (version 1.9) (http://pngu.mgh.harvard.edu/purcell/plink/ (accessed on 10 October 2021)) [29].

## 3. Results

In total, 23 SNPs in 17 genes were assessed in either the Czech or the Irish cohorts or both. The ORs and 95% CIs for their associations with CRC risk are presented in Table 2 and Table S2, respectively, for each study.

In the larger Czech cohort, a significant association with an increased CRC risk was only observed for SNP rs4802034 in the *SELENOV* gene (recessive model OR = 2.14; 95% CI: 1.23, 3.72, *p* = 0.007; additive model OR = 1.44; 95% CI: 1.08, 1.91, *p* = 0.01; Table 2).

In the Irish cohort, significant associations with higher CRC risks were observed for variants in two selenoprotein genes: rs5859 in *SELENOF* (dominant model OR = 1.54; 95% CI: 1.10, 2.15, *p* = 0.012; additive model OR = 1.49; 95% CI: 1.12, 1.98, *p* = 0.006) and rs2972994 in *SELENOP* (dominant model OR = 1.44; 95% CI: 1.01, 2.05, *p* = 0.044), with similar risk estimates for advanced colorectal neoplasia (i.e., tumors and adenomas combined). An association with a decreased disease risk in the dominant genetic model was observed for rs34713741 in *SELENOS* with CRC (OR = 0.73; 95% CI: 0.53, 0.99, *p* = 0.049), advanced colorectal neoplasia (*p* = 0.011), and CRA (i.e., advanced adenomas including those with HGD, *p* = 0.046), although this variant was borderline for HWE in cases (*p* = 0.93). Significant associations were also observed with an increased risk of advanced colorectal neoplasia and CRA for rs4659382 in *SELENON* and rs2275129 in *SEPHS1*. It was also observed that rs3813498 in *FOXO3* was associated (*p* = 0.011) with a decreased risk of colorectal neoplasia in the dominant genetic model with OR = 0.69 and CI: 0.52, 0.92. The significant findings for colorectal neoplasia categories are shown in Table 3, while all the analyses for CRC are presented in Table S2.

None of the SNP associations in either cohort retained significance following multiple test corrections. Tables S3 and S4 contain the ORs, 95% CIs, *p*-values, and FDR corrections for the full stratified analyses of the Irish and Czech cohorts, respectively. In the Irish cohort, these analyses were stratified based on neoplasia subtype (i.e., polyp, adenoma, HGD, cancer) with adjustment for age and sex; the Czech cohort was stratified based on sex and cancer subsite (i.e., colon, rectum) with adjustment for age and sex.

There were no major findings in the Czech stratified analyses by major anatomical subsite. Only rs2275129 (*SEPSH1*) was nominally associated with increased rectal cancer risk, but the raw *p*-value was just under the significance threshold (*p* = 0.044), and this may well have been a chance finding due to the multiple comparisons (Table S4).

## 4. Discussion

In this study, we observed limited associations for colorectal neoplasia risk with polymorphisms in selenoprotein (and related Se metabolism) genes for the studied populations from the Czech Republic and Ireland. Nominally significantly increased CRC risks were observed for rs4802034-*SELENOV* in the Czech cohort (*p* = 0.007) and rs5859-*SELENOF* (*p* = 0.006) and rs2972994-*SELENOP* (*p* = 0.044) in the Irish cohort, while there was a decreased CRC risk observed for rs34713741 in *SELENOS* in the latter group.

In the stratified analyses by cancer anatomic location and neoplasia type, only rs2275129 in *the SEPSH1* gene was associated with an increased risk of rectal cancer in the Czechs. For neoplasia stratification in the Irish cohort, several variants in selenoprotein and Se metabolism genes, i.e., rs5859-*SELENOF*, rs4659382-*SELENON*, rs2972994-*SELENOP*, and rs2275129-*SEPHS1*, were associated with an increased risk of advanced colorectal neoplasia, whereas *SELENOS rs34713741* was associated with a decreased risk of advanced colorectal neoplasia.

While none of the findings retained significance after FDR multiple-testing adjustment, such corrections could be considered over-stringent given that all our analyses were planned a priori, based on our stated hypothesis, and with a modest number of related Se pathway genetic variants previously implicated in CRC development.

The association of rs4802034 in the *SELENOV* gene with increased CRC risk in the Czechs (*p* = 0.007) was also observed for the recessive genetic model (*p* = 0.021) in the EPIC study [10]. Its protein product has a poorly characterized function, particularly regarding its role in influencing colorectal function and the development of CRC [30]. As recently shown in murine models, *SELENOV* gene variation may alter the response to the endoplasmic reticulum (ER) and oxidative stresses [31], and these processes are also implicated in colorectal carcinogenesis [10].

The current study demonstrated that *SELENOF* rs5859 was associated with an increased risk of CRC and all advanced colorectal neoplasia in the Irish cohort. *SELENOF* is thought to play a role in ER protein folding and quality control [32] and has been implicated in prostate cancer and survival after CRC diagnosis in previous studies [33,34]. Two-loci interactions of rs5859 with several different SNPs in *SELENOP* showed significant associations with altered CRC risk in a previous Czech study by Méplan et al. [11], although the sample sizes for these analyses were small, and the variant was not associated with CRC risk alone. Our observation that rs2972994-*SELENOP* was associated with an increased risk of CRC and all colorectal neoplasia in the Irish cohort (*p* = 0.044) aligned with previous studies conducted by Peters et al. [35,36] on advanced CRA. The SELENOP protein is synthesized in the liver and is primarily responsible for the transport of Se to distal tissue, although it also has antioxidant properties in the extracellular space [37]. Genetic variation in the *SELENOP* gene may contribute to colorectal carcinogenesis by reducing the protein’s antioxidant properties and reducing the colonic tissue bioavailability of Se [38]. Méplan et al. [11] observed a sex-specific differential risk direction for rs2972994-*SELENOP,* in that rs2972994-*SELENOP* was not significantly associated with CRC risk for men and women combined, but the authors observed that men with one T allele variant were significantly associated with a higher risk of CRC whereas, in women, the risk of CRC was significantly decreased. This may point to complex interactions between the hierarchy of Se metabolism and sex, contributing to varying effects of this SNP on CRC development risk. The rs2972994 is located on the 44321bp 3′ of STP in proximity to the selenocysteine insertion sequence in the 3′ promoter region of the *SELENOP* gene [36]. Although the range of functions of the *SELENOP* gene is not fully understood, polymorphic antisense transcripts influence gene expression by regulating post-transcriptional modifications [28]. The rs2972994-*SELENOP* variant is expected to interrupt the STAF transcription factor binding site of the Matlnspector-based *SELENOP* gene [39].

*SELENON* encodes an ER-localized glycoprotein that is involved in the oxidative protection and regulation of calcium homeostasis; it has thus been implicated in muscle development and function [40]. Hughes et al. [33] found that this protein was significantly (*p* = 0.001) downregulated in CRC tumor tissues. *SELENON* rs4659382 was nominally associated (*p* = 0.035) in the additive model with an increased risk of CRA and with all advanced neoplasia in the Irish cohort, as observed for CRC (dominant model: *p* = 0.005) in the EPIC study [10].

*SEPHS1* encodes selenophosphate synthetase 1. While this is not itself a selenoprotein, it plays a crucial role in selenoprotein synthesis by using selenide and ATP to synthesize selenophosphate, the key Se donor in the production of selenocysteine [41]. SNP variants may decrease the efficiency of this enzyme and reduce selenoprotein production, thus contributing to an increased risk of CRC development. We observed that rs2275129 in *SEPHS1* was associated with increased colorectal neoplasia risk in the Irish population, although not CRC risk alone, as was shown in the previous EPIC study [10].

*SELENOS* plays a vital role in ER stress response and inflammation control. Interestingly, *SELENOS*-rs34713741 (a functional promoter variant) showed nominal significance for a decreased association with CRA risk in the Irish cohort, with or without small polyps in the control group. Méplan et al. [11] and Li et al. [42] both found this SNP to be associated with an increased risk of CRC. It also appears in our study that this SNP is significantly associated with a decreased risk of advanced colorectal neoplasia in the dominant model only (*p* = 0.011), whereas the point estimates for the recessive and additive model yielded non-significant inverse associations with CRA risk. This may simply have resulted from type 1 errors as the raw *p* values were close to the significance cut-off. However, it may also reflect unknown genotyping errors for this SNP or a possible chromosomal crossover event in the tagged variant, which may also explain why the SNP was not in HWE in the Irish cases (with polyps excluded). Alternatively, although this is unlikely, there may be an interaction between this selenoprotein gene variant and the hierarchy of Se metabolism that led to differences in risk directions. *SELENOS* encodes a protein involved in the unfolded protein response, shuttling misfolded proteins from the ER lumen to the cytosol where they are degraded by ubiquitin-dependent mechanisms. It also influences inflammatory signaling pathways, which reduces ER stress and may also affect colonic cell growth [7,43]. Curan et al. [43] conducted a functional analysis of rs34713741 and provided evidence that the A allele significantly altered the expression of *SELENOS* after exposure to ER stress agents (*p* = 6 × 10^−5^). Furthermore, the downregulation of the *SELENOS* gene in macrophage cells by short interfering RNA enhanced the IL-6 and TNF-a (inflammatory cytokines) release, depicting its importance as an inflammation mediator. Hence, as for *SELENOS*, functional variants in this gene may also impact ER and inflammatory stress responses implicated in CRC development.

Among the wider Se pathway genes, *FOXO3* plays a vital role as a central transcription factor that mediates various pathological and physiological processes including progression of the cell cycle, apoptosis, and survival [44,45]. We demonstrated that rs3813498 in *FOXO3* was associated with a decreased risk of all colorectal neoplasia (OR dominant model: 0.69; CI: 0.52, 0.92, *p* = 0.011) in the Irish cohort, as previously observed in EPIC (OR: 0.79; CI: 0.67, 0.92, *p* = 0.003).

Several studies in different regions of the world suggest that genetic variation in different selenoproteins can influence CRC risk [11,12,41] and that this can be modified by Se status [10,14,46]. This association with selenoprotein variants and CRC risk is thought to be more apparent in areas of suboptimal Se availability; one possible explanation for these differences is that a higher intake of Se may counteract the decreased functional efficiency of selenoprotein synthesis (and possible subsequent modified CRC risk) caused by selenoprotein gene variants and the hierarchical regulation of selenoprotein biosynthesis [11,44].

A limitation of our study was the absence of comprehensive Se status data (not available for the Czechs and only available for a subset of the Irish study). Thus, we lacked the power to perform a rigorous SNPxSe status interaction analysis.

The Irish study was analyzed using different case–control allocations, with generally no major impact on the results when small non-advanced polyps were excluded or included from the case or control groups. For significant associations, there seemed to be more consistent associations observed with polyps in the control group or omitted from the analysis. It is not possible to make a definitive conclusion on the effects of polyps on cancer associations since none of these associations were significant following multiple testing corrections. Many CRC case–control studies either are unable to account for the presence of these small polyps, or deliberately include them in the control group due to their common occurrence in controls of an appropriately similar age range to the cases (as for the Czech cohort in this study). A strength of this study is its consideration of these issues and the inference from the results that it is reasonable to include small polyps in control groups, though it may be more robust to exclude them from analyses or consider them a distinct pathology group.

## 5. Conclusions

Overall, the results of this study add to the finding from our previous studies [10,11] that the risk of CRC may be modified by selenoprotein genotypes, although the context of the contribution of this genetic variation appears complicated and divergent in different study settings. Individuals with those selenoprotein genotypes associated with increased colorectal neoplasia risk may benefit from increasing their Se status.

## Figures and Tables

**Table 1:** Selected baseline characteristics of the colorectal neoplasia cases and the controls in the Czech and Irish studies.

Study Characteristics	Czech Republic		Republic of Ireland	
	CRC Cases	Controls	CRC Cases	Controls
N (%)	718 (52.64)	646 (47.36)	241 (26.5)	461 (50.6)
Age	64.13 ± 12.01	50.73 ± 13.62	65.75 ± 11.50	52.05 (±11.31)
Sex (males, %)	432 (60.18)	365 (56.50)	156 (64.7)	210 (45.6)
Smoking status				
Non-smokers (%)	414 (57.66)	395 (61.15)		
Ex-smokers (%)	186 (25.91)	111 (17.18)		
Current smokers (%)	118 (16.43)	140 (21.67)		
Mean BMI (kg/m^2^) ± SD	27.17 ± 5.02	26.01 ± 4.62		
Alcohol Consumption (%)	27.17 ± 5.02	26.01 ± 4.62		
Yes (%)	182 (25.3)	202 (31.3)		
No (%)	128 (17.8)	129 (19.9)		
Positive Family History of Cancer				
Yes (%)	275 (38.3)	284 (43.9)		
No (%)	359 (50.0)	308 (47.7)		
Colorectal cancer				
Colon cancer (%)	487 (67.83)		129 (53.53)	
Rectal cancer (%)	231 (32.17) ^1^		114 (47.30)	
T staging n (T1/T2/T3/T4)	26/116/406/91		10/9/20/7	
N staging n (N0/N1/N2)	353/188/87		37/5/4	
M staging n (M0/M1)	513/117		9/5	
Stage (I/II/III/IV)	92/399/103/3		13/20/9/5	
Other neoplasia (%)			209 (22.9)	
Polyps ^2^ (%)			110 (52.6)	
Adenoma ^3^ (%)			75 (35.9)	
Adenomas with high grade dysplasia ^4^ (%)			24 (11.5)	

^1^ Percentages do not add up to 100% due to missing values. ^2^ Polyps were generally hyperplastic and less than 2 mm. ^3^ Adenomas were all >10 mm and/or with villous or tubulovillous components. ^4^ Adenomas with high-grade dysplasia were advanced adenomas with at least 5% HGD. Abbreviations: CRC, colorectal cancer; TNM staging,: tumor stage, regional lymph node involvement, and distant metastasis; BMI, body mass index.

**Table 2:** Associations of the selenoprotein gene variants with colorectal cancer risk in the Czech cohort.

Gene	SNP	Genotype	Cases	Controls	OR	95% CI	*p* *	FDR
*GPX1*	rs9818758	GG/GA/AA	497/201/19	437/171/19				
		Dominant (GG vs. GA + AA)	717	627	1.09	0.84, 1.44	0.51	0.86
		Recessive (AA vs. GG + GA)	717	627	0.88	0.41, 1.91	0.74	0.94
		Additive	717	627	0.95	0.65, 1.40	0.81	0.94
*GPX4*	rs2074451	GG/GT/TT	221/350/141	191/324/108				
		Dominant (GG vs. GT + TT)	712	623	1.09	0.83, 1.43	0.54	0.86
		Recessive (TT vs. GG + GT)	712	623	1.25	0.91, 1.73	0.17	0.86
		Additive	712	623	1.13	0.94, 1.36	0.19	0.86
*GPX5*	rs445870	AA/AG/GG	388/271/48	336/236/55				
		Dominant (AA vs. AG + GG)	707	627	1.00	0.78, 1.29	0.99	0.99
		Recessive (GG vs. AA + AG)	707	627	0.96	0.59, 1.54	0.86	0.94
		Additive	707	627	0.98	0.77, 1.25	0.87	0.94
*SELENOM*	rs11705137	TT/TC/CC	224/350/135	190/290/137				
		Dominant (TT vs. TC + CC)	709	617	0.91	0.69, 1.19	0.49	0.86
		Recessive (CC vs. TT + TC)	709	617	0.86	0.63, 1.17	0.33	0.86
		Additive	709	617	0.91	0.76, 1.09	0.29	0.86
*SELENON*	rs11247710	CC/CG/GG	229/370/115	209/313/104				
		Dominant (CC vs. CG + GG)	714	626	1.09	0.84, 1.43	0.51	0.86
		Recessive (GG vs. CC + CG)	714	626	0.87	0.62, 1.22	0.42	0.86
		Additive*	714	626	0.97	0.80, 1.18	0.45	0.86
*SELENON*	rs4659382	CC/CG/GG	447/240/30	386/214/31				
		Dominant (CC vs. CG + GG)	717	631	1.02	0.79, 1.32	0.86	0.94
		Recessive (GG vs. CC + CG)	717	631	0.77	0.42, 1.41	0.41	0.86
		Additive	717	631	0.89	0.65, 1.21	0.45	0.86
*SELENOV*	rs4802034	CC/CT/TT	386/277/48	334/262/27				
		Dominant (CC vs. CT + TT)	711	623	1.04	0.81, 1.33	0.79	0.94
		Recessive (TT vs. CC + CT)	711	623	2.14	1.23, 3.72	**0.007**	0.19
		Additive	711	623	1.44	1.08, 1.91	**0.01**	0.19
*TXNRD1*	rs11111979	GG/GC/CC	203/328/174	171/316/139				
		Dominant (GG vs. GC + CC)	705	626	0.93	0.70, 1.23	0.59	0.86
		Recessive (CC vs. GG + GC)	705	626	1.08	0.81, 1.45	0.61	0.86
		Additive	705	626	1.00	0.84, 1.19	0.98	0.99
*TXNRD1*	rs7953266	TT/TC/CC	256/323/133	210/312/105				
		Dominant (TT vs. TC + CC)	712	627	0.86	0.66, 1.11	0.24	0.86
		Recessive (CC vs. TT + TC)	712	627	1.01	0.72, 1.39	0.97	0.99
		Additive	712	627	0.95	0.79, 1.14	0.61	0.86
*BAX*	rs4645887	TT/TA/AA	261/334/119	208/322/94				
		Dominant (TT vs. TA + AA)	714	624	0.89	0.69, 1.16	0.39	0.86
		Recessive (AA vs. TT + TA)	714	624	1.29	0.91, 1.83	0.16	0.86
		Additive	714	624	1.07	0.88, 1.30	0.48	0.86
*FRZB*	rs17265803	TT/TC/CC	521/179/14	477/140/10				
		Dominant (TT vs. TC + CC)	714	627	1.22	0.92, 1.63	0.18	0.86
		Recessive (CC vs. TT + TC)	714	627	1.54	0.61, 3.87	0.36	0.86
		Additive	714	627	1.27	0.79, 2.01	0.31	0.86
*FOXO3*	rs3813498	TT/TC/CC	477/207/28	421/180/26				
		Dominant (TT vs. TC + CC)	712	627	1.08	0.82, 1.41	0.59	0.86
		Recessive (CC vs. TT + TC)	712	627	1.08	0.56, 2.07	0.83	0.94
		Additive	712	627	1.05	0.75, 1.46	0.78	0.94
*SEPHS1*	rs2275129	GG/GC/CC	224/339/150	190/326/112				
		Dominant (GG vs. GC + CC)	713	628	0.93	0.71, 1.23	0.62	0.86
		Recessive (CC vs. GG + GC)	713	628	1.36	0.99, 1.88	0.06	0.78
		Additive	713	628	1.11	0.92, 1.33	0.28	0.86

SNP, single nucleotide polymorphism; OR, odds ratio; CI, confidence interval. * *p*-value obtained from logistic regression model (additive, dominant, recessive), adjusted by age, sex, smoking, BMI, and alcohol consumption; *p*-value considered significant at < 0.05 and noted in bold.

**Table 3:** The significant associations between SNPs and colorectal neoplasia risk for different subsets of the stratified analysis in the Irish cohort.

Gene	SNP	Case/Control	Genotype	Cases	Controls	OR	95%CI	*p* *	FDR
*SELENOF*	rs5859	Cancer/Control	CC/CT/TT	129/93/14	239/118/11				
			Dominant (CC vs. CT + TT)	236	368	1.54	1.10, 2.15	**0.012**	0.50
			Recessive (TT vs. CT + CC)	236	368	2.05	0.91, 4.59	0.082	0.74
			Additive	236	368	1.49	1.12, 1.98	**0.006**	0.50
*SELENOF*	rs5859	Cancer/Control + Polyp	CC/CT/TT	129/93/14	305/156/16				
			Dominant (CC vs. CT + TT)	236	477	1.47	1.07, 2.02	**0.017**	0.50
			Recessive (TT vs. CT + CC)	236	477	1.82	0.87, 3.79	0.111	0.79
			Additive	236	477	1.42	1.09, 1.86	**0.010**	0.50
*SELENOF*	rs5859	Adenoma + HGD + Cancer/Control	CC/CT/TT	185/129/20	239/118/11				
			Dominant (CC vs. CT + TT)	334	368	1.42	1.07, 1.88	**0.016**	0.50
			Recessive (TT vs. CT + CC)	334	368	1.94	0.94, 4.00	0.072	0.71
			Additive	334	368	1.39	1.09, 1.78	**0.008**	0.50
*SELENOF*	rs5859	Adenoma + HGD + Cancer/Control + Polyp	CC/CT/TT	185/129/20	305/156/16				
			Dominant (CC vs. CT + TT)	334	477	1.43	1.07, 1.90	**0.014**	0.50
			Recessive (TT vs. CT + CC)	334	477	1.84	0.94, 3.59	**0.008**	0.50
			Additive	334	477	1.39	1.09, 1.77	**0.007**	0.50
*SELENON*	rs4659382	Adenoma + HGD + Cancer/Control	TT/TA/AA	248/164/30	133/76/5				
			Dominant (TT vs. TA +AA)	442	214	1.28	0.92, 1.79	0.142	0.68
			Recessive (AA vs. TA + TT)	442	214	3.04	1.16, 7.96	**0.023**	0.80
			Additive	442	214	1.36	1.02, 1.80	**0.035**	0.57
*SELENON*	rs4659382	Adenoma + HGD/Control	TT/TA/AA	49/41/7	133/76/5				
			Dominant (TT vs. TA +AA)	97	214	1.61	0.99, 2.61	0.054	0.69
			Recessive (AA vs. TA + TT)	97	214	3.25	1.01, 10.5	**0.049**	0.69
			Additive	97	214	1.64	1.08, 2.48	**0.019**	0.50
*SELENON*	rs4659382	Polyp + Adenoma + HGD/Control	TT/TA/AA	110/80/16	133/76/5				
			Dominant (TT vs. TA +AA)	206	214	1.43	0.97, 2.11	0.069	0.50
			Recessive (AA vs. TA + TT)	206	214	3.52	1.26, 9.79	**0.016**	0.72
			Additive	206	214	1.51	1.08, 2.10	**0.015**	0.50
*SELENOP*	rs2972994	Cancer/Control + Polyp	GG/GA/AA	58/123/81	148/221/102				
			Dominant (GG vs. GA + AA)	240	471	1.44	1.01, 2.05	**0.044**	0.69
			Recessive (AA vs. GA + GG	240	471	1.18	0.82, 1.70	0.378	0.89
			Additive	240	471	1.22	0.98, 1.51	1.227	0.69
*SELENOP*	rs2972994	Adenoma + HGD + Cancer/Control + Polyp	GG/GA/AA	85/136/87	148/221/102				
			Dominant (GG vs. GA + AA)	308	471	1.37	1.00, 1.87	**0.049**	0.69
			Recessive (AA vs. GA + GG	308	471	1.25	0.89, 1.73	0.182	0.80
			Additive	308	471	1.22	1.00, 1.49	**0.043**	0.69
*SELENOS*	rs34713741	Cancer/Control + Polyp	CC/CT/TT	136/77/22	236/207/29				
			Dominant (CC vs. CT + TT)	235	472	0.73	0.53, 0.99	**0.049**	0.69
			Recessive (TT vs. CT + CC)	235	472	1.58	0.88, 2.81	0.122	0.80
			Additive	235	472	0.89	0.69, 1.14	0.352	0.89
*SELENOS*	rs34713741	Adenoma+HGD/Control	CC/CT/TT	59/31/5	183/159/20				
			Dominant (CC vs. CT + TT)	95	362	0.62	0.39, 0.99	**0.046**	0.75
			Recessive (TT vs. CT + CC)	95	362	0.95	0.35, 2.60	0.921	0.99
			Additive	95	362	0.71	0.48, 1.05	0.088	0.69
*SELENOS*	rs34713741	Adenoma + HGD/Control + Polyp	CC/CT/TT	59/31/5	236/207/29				
			Dominant (CC vs. CT + TT)	95	472	0.61	0.39, 0.96	**0.032**	0.71
			Recessive (TT vs. CT + CC)	95	472	0.85	0.32, 2.25	0.741	0.94
			Additive	95	472	0.69	0.47, 1.01	0.060	0.68
*SEPSH1*	rs2275129	Adenoma + HGD + Cancer/Control	GG/GC/CC	78/136/87	56/116/44				
			Dominant (GG vs. GC + CC)	301	216	0.96	0.66, 1.40	0.831	0.85
			Recessive (CC vs. GC + GG)	301	216	1.52	1.02, 2.25	**0.039**	0.99
			Additive	301	216	1.14	0.91, 1.43	0.263	0.69
*SEPSH1*	rs2275129	Adenoma + HGD + Cancer/Control + Polyp	GG/GC/CC	78/136/87	87/165/71				
			Dominant (GG vs. GC + CC)	301	323	1.05	0.74, 1.51	0.772	0.80
			Recessive (CC vs. GC + GG)	301	323	1.44	1.00, 2.07	**0.047**	0.95
			Additive	301	323	1.17	0.94, 1.45	0.169	0.69
*SEPSH1*	rs2275129	Polyp + Adenoma + HGD/Control	GG/GC/CC	51/91/59	56/116/44				
			Dominant (GG vs. GC + CC)	201	216	1.03	0.66, 1.59	0.897	0.80
			Recessive (CC vs. GC + GG)	201	216	1.62	1.04, 2.55	**0.034**	0.99
			Additive	201	216	1.21	0.92, 1.58	0.171	0.68
*SEPSH1*	rs2275129	Adenoma + HGD/Control	GG/GC/CC	20/42/32	56/116/44				
			Dominant (GG vs. GC + CC)	94	216	1.29	0.72, 2.31	0.382	0.68
			Recessive (CC vs. GC + GG)	94	216	2.02	1.17, 3.46	0.011	0.89
			Additive	94	216	1.46	1.03, 2.08	**0.035**	0.50
*SEPSH1*	rs2275129	Adenoma + HGD/Control + Polyp	GG/GC/CC	20/42/32	87/165/71				
			Dominant (GG vs. GC + CC)	94	323	1.36	0.79, 2.37	0.270	0.68
			Recessive (CC vs. GC + GG)	94	323	1.83	1.11, 3.02	**0.018**	0.85
			Additive	94	323	1.43	1.03, 1.98	**0.034**	0.50
*FOXO3*	rs3813498	Adenoma + HGD + Cancer/Control + Polyp	TT/TC/CC	221/103/14	212/102/8				
			Dominant (TT vs. TC + CC)	338	322	0.69	0.52, 0.92	**0.011**	0.50
			Recessive (CC vs. TC + TT)	338	322	1.70	0.70, 4.09	0.241	0.83
			Additive	338	322	1.07	0.81, 1.42	0.621	0.92

Polyps were generally hyperplastic and less than 2 mm. Adenomas were all >10 mm and/or with villous or tubulovillous components. Adenomas with high-grade dysplasia were advanced adenomas with at least 5% HGD. SNP, single nucleotide polymorphism; OR, odd ratio; CI, confidence interval; Ca/Co, case/control; HGD, high-grade dysplasia. * *p*-value obtained from logistic regression model (additive, dominant, recessive), adjusted by age; *p*-value considered significant at <0.05 and noted in bold.

## Data Availability

Not applicable.

## Supplementary Materials

The following supporting information can be downloaded at: https://www.mdpi.com/article/10.3390/nu14132718/s1, Table S1: Allele frequencies for SNPs observed in both the Czech and Irish study populations. Table S2: Associations of gene variants with colorectal cancer risk in the Irish cohort; Table S3: OR’s, 95% CIs, *p*-values and FDR corrections for the full stratified analyses of the Irish cohorts.; Table S4: OR’s, 95% CIs, *p*-values and FDR corrections for the full stratified analyses of the Czech Republic cohort.
