# Supplementary material for: The Associations of Selenoprotein Genetic Variants with the Risks of Colorectal Adenoma and Colorectal Cancer: Case–Control Studies in Irish and Czech Populations
Source: Nutrients. 2022 Jun 29;14(13):2718. doi: 10.3390/nu14132718 (PMC9268344; doi:10.3390/nu14132718)
Supplement: Supplementary file 1 [file nutrients-14-02718-s001.zip › S. Table S1 HWE DH May 2022.pdf]

Table S1: Allele frequencies for SNPs observed in both the Czech and Irish study populations.

Republic of Ireland						Czech Republic					
**Genes**	**SNP**	**Allele 1/Allele 2**	**Cases**	**Control**	**P-Value**	**Genes**	**SNP**	**Allele 1/Allele 2**	**Cases**	**Control**	**P-Value**
GPX1	rs1050450	T/C	141/305	237/481	0.57	*GPX1*	rs9818758	A/G	239/1195	209/1045	0.92
GPX3	rs8177426	A/G	91/373	129/599	0.06	*GPX4*	rs2074451	T/G	632/792	540/706	0.87
GPX3	rs8177447	T/C	85/375	124/602	0.14	*GPX5*	rs445870	G/A	367/1047	346/908	0.30
GPX4	rs2074451	T/G	220/198	194/218	0.60	*SELENOM*	rs11705137	C/T	620/798	564/670	1.00
GPX4	rs713041	T/C	203/233	304/374	0.03	*SELENON*	rs4659382	G/C	300/1134	276/986	1.00
GPX5	rs445870	G/A	132/310	135/295	0.07	*SELENON*	rs1124771	G/C	600/828	521/731	0.11
SELENOF	rs5859	T/C	117/339	140/596	0.69	*SELENOV*	rs4802034	T/C	373/1049	316/930	0.05
SELENOM	rs11705137	T/C	215/207	203/215	1.00	*SEPSH1*	rs2275129	C/G	639/787	550/706	0.89
SELENON	rs4659382	G/C	105/337	86/342	0.60	*TXNRD1*	rs11111979	C/G	676/734	594/658	0.30
SELENON	rs1124771	G/C	189/219	185/241	0.10	*TXNRD1*	rs7953266	C/T	594/840	522/732	0.61
SELENOP	rs2972994	A/G	234/230	330/396	0.23	*BAX*	rs4645887	A/T	572/856	510/738	0.55
SELENOP	rs7579	A/G	122/330	176/552	0.71	*FOXO3*	rs3813498	C/T	263/1161	232/1022	0.15
SELENOS	rs34713741	T/C	115/341	199/525	0.93	*FRZB*	rs17265803	T/C	207/1221	160/1094	0.37
SEPSH1	rs2275129	C/G	187/199	204/228	0.60						
SOD2	rs4880	T/C	229/227	360/372	0.90						
TXNRD1	rs11111979	C/G	219/231	199/229	0.04						
TXNRD2	rs9605031	T/C	117/337	167/565	0.53						
BAX	rs4645887	A/T	167/273	173/249	0.62						
FOXO3	rs3813498	C/T	85/369	81/345	0.90						
FRZB	rs17265803	C/T	53/413	45/385	0.05						

p-value indicating the Hardy Weinberg Equilibrium significance > 0.05 for cases and controls combined
SNP-Single Nucleotide Polymorphism
