# Supplementary material for: The Associations of Selenoprotein Genetic Variants with the Risks of Colorectal Adenoma and Colorectal Cancer: Case–Control Studies in Irish and Czech Populations
Source: Nutrients. 2022 Jun 29;14(13):2718. doi: 10.3390/nu14132718 (PMC9268344; doi:10.3390/nu14132718)
Supplement: Supplementary file 1 [file nutrients-14-02718-s001.zip › S. Table S2 May 2022 DH.pdf]

Table S2: Associations of gene variants with colorectal cancer risk in the Irish cohort.

Gene	SNP	Genotype	Cases	Control	OR	95%CI	P*	FRD
GPX1	rs1050450	CC/CT/TT	113/91/26	162/157/40				
		Dominant (CC vs CT+TT)	230	359	0.85	0.61, 1.18	0.342	0.74
		Recessive (TT vs CT+CC)	230	359	1.11	0.60, 1.72	0.951	0.95
		Additive	230	359	0.92	0.72, 1.18	0.499	0.74
GPX3	rs8177426	GG/GA/AA	151/84/5	243/113/8				
		Dominant (GG vs GA + AA)	240	364	1.18	0.84, 1.66	0.332	0.74
		Recessive (AA vs GA + GG	240	364	0.95	0.31, 2.93	0.924	0.95
		Additive	240	364	1.14	0.84, 1.56	0.394	0.74
GPX3	rs8177447	CC/CT/TT	155/78/5	247/108/8				
		Dominant (CC vs CT+TT)	238	363	1.14	0.81, 1.61	0.457	0.74
		Recessive (TT vs CT+CC)	238	363	0.95	0.31, 2.95	0.932	0.95
		Additive	238	363	1.11	0.81, 1.52	0.517	0.74
GPX4	rs2074451	TT/TG/GG	65/107/52	46/102/58				
		Dominant (TT vs TG + GG)	224	206	1.08	0.75, 1.56	0.681	0.82
		Recessive (GG vs TG + GG)	224	206	1.15	0.77, 1.72	0.493	0.74
		Additive	224	206	1.08	0.86, 1.36	0.512	0.74
GPX4	rs713041	CC/CT/TT	68/103/54	108/103/73				
		Dominant (CC vs CT+TT)	225	284	0.76	0.52, 1.13	0.177	0.68
		Recessive (TT vs CT+CC)	225	284	0.95	0.55, 1.64	0.864	0.95
		Additive	225	284	0.86	0.65, 1.14	0.288	0.74
GPX5	rs445870	AA/AG/GG	113/104/19	108/79/28				
		Dominant (AA vs AG +GG)	236	215	0.87	0.59, 1.29	0.489	0.74
		Recessive (GG vs AG + AA)	236	215	2.25	0.69, 7.29	0.176	0.68
		Additive	236	215	0.97	0.69, 1.36	0.870	0.95
SELENOF	rs5859	CC/CT/TT	129/93/14	239/118/11				

		Dominant (CC vs CT+TT)	236	368	1.54	1.10, 2.15	**0.012**	0.35
		Recessive (TT vs CT+CC)	236	368	2.05	0.91, 4.59	0.082	0.62
		Additive	236	368	1.49	1.12, 1.98	**0.006**	0.35
SELENOM	rs11705137	CC/CT/TT	47/125/53	52/111/46				
		Dominant (CC vs CT+TT)	225	209	1.29	0.93, 1.81	0.123	0.67
		Recessive (TT vs CT+CC)	225	209	1.09	0.57, 2.12	0.789	0.89
		Additive	225	209	1.19	0.92, 1.56	0.179	0.68
SELENON	rs11247710	CC/CG/GG	67/104/44	67/104/48				
		Dominant (CC vs CG+GG)	215	219	1.16	0.77, 1.74	0.476	0.74
		Recessive (GG vs CG+CC)	215	219	1.08	0.68, 1.71	0.749	0.86
		Additive	215	219	1.09	0.84, 1.41	0.521	0.74
SELENON	rs4659382	CC/CG/GG	138/84/14	133/76/5				
		Dominant (CC vs CG+GG)	236	214	1.17	0.79, 1.70	0.427	0.74
		Recessive (GG vs CG+CC)	236	214	2.64	0.93, 7.45	0.067	0.62
		Additive	236	214	1.25	0.90, 1.73	0.181	0.68
SELENOP	rs2972994	GG/GA/AA	58/123/59	114/168/81				
		Dominant (GG vs GA + AA)	240	363	1.44	0.99, 2.08	**0.044**	0.62
		Recessive (AA vs GA + GG	240	363	0.14	0.77, 1.67	0.518	0.74
		Additive	240	363	1.20	0.96, 1.51	0.112	0.67
SELENOP	rs7579	GG/GA/AA	123/93/17	208/136/20				
		Dominant (GG vs GA + AA)	233	364	1.19	0.86, 1.66	0.297	0.74
		Recessive (AA vs GA + GG	233	364	1.35	0.69, 2.64	0.375	0.74
		Additive	233	364	1.18	0.90, 1.54	0.231	0.74
SELENOS	rs34713741	CC/CT/TT	136/77/22	183/159/20				
		Dominant (CC vs CT+TT)	235	362	0.74	0.53, 1.04	0.080	0.62
		Recessive (TT vs CT+CC)	235	362	1.77	0.94, 3.31	0.076	0.62
		Additive	235	362	0.91	0.70, 1.19	0.505	0.74
SEPHS1	rs2275129	GG/GC/CC	58/94/55	56/116/44				

		Dominant (GG vs GC + CC)	207	216	0.89	0.59, 1.38	0.628	0.79
		Recessive (CC vs GC + GG)	207	216	1.41	0.89, 2.22	0.133	0.67
		Additive	207	216	1.09	0.83, 1.42	0.552	0.77
SOD2	rs4880	CC/CT/TT	55/122/59	99/174/93				
		Dominant (CC vs CT+TT)	236	366	1.22	0.83, 1.78	0.304	0.74
		Recessive (TT vs CT+CC)	236	366	0.98	0.67, 1.43	0.910	0.95
		Additive	236	366	1.07	0.85, 1.34	0.575	0.78
TXNRD1	rs11111979	GG/GC/CC	68/107/65	67/95/52				
		Dominant (GG vs GC + CC)	240	214	1.15	0.77, 1.73	0.489	0.74
		Recessive (CC vs GC + GG)	240	214	1.16	0.76, 1.77	0.499	0.74
		Additive	240	214	1.11	0.87, 1.42	0.411	0.74
TXNRD2	rs9605031	CC/CT/TT	127/91/16	122/121/23				
		Dominant (CC vs CT+TT)	234	266	1.25	0.80, 1.97	0.323	0.74
		Recessive (TT vs CT+CC)	234	266	1.09	0.69, 1.71	0.701	0.83
		Additive	234	266	1.13	0.85, 1.49	0.394	0.74
BAX	rs4645887	TT/TA/AA	89/115/31	67/115/29				
		Dominant (TT vs TA +AA)	235	211	0.70	0.45, 1.09	0.441	0.74
		Recessive (AA vs TA + TT)	235	211	0.77	0.49, 1.19	0.241	0.74
		Additive	235	211	0.79	0.61, 1.04	0.094	0.62
FOXO3	rs3813498	TT/TC/CC	162/70/10	136/73/4				
		Dominant (TT vs TC + CC)	242	213	1.09	0.76, 1.59	0.618	0.79
		Recessive (CC vs TC + TT)	242	213	0.59	0.32, 1.08	0.087	0.62
		Additive	242	213	0.94	0.72, 1.24	0.677	0.82
FRZB	rs17265803	TT/TC/CC	192/55/1	171/43/1				
		Dominant (TT vs TC + CC)	248	215	1.13	0.73, 1.77	0.581	0.78
		Recessive (CC vs TC + TT)	248	215	0.87	0.05, 13.94	0.919	0.95
		Additive	248	215	1.12	0.73, 1.73	0.604	0.79

SNP=Single Nucleotide Polymorphism; OR=Odd Ratio; CI=Confidence Interval

Case=Cancer patients; Control=Healthy individuals excluding polyp
*p-values obtained from logistic regression model (Additive, Dominant, Recessive); ***p*-value considered significant at < 0.05 and noted in bold.**
FDR- False Discovery Rate obtained by the Benjamini–Hochberg procedure.
