# Supplementary material for: The Associations of Selenoprotein Genetic Variants with the Risks of Colorectal Adenoma and Colorectal Cancer: Case–Control Studies in Irish and Czech Populations
Source: Nutrients. 2022 Jun 29;14(13):2718. doi: 10.3390/nu14132718 (PMC9268344; doi:10.3390/nu14132718)
Supplement: Supplementary file 1 [file nutrients-14-02718-s001.zip › S. Table S3 May 2022 DH.pdf]

**Table S3:** ORs, 95% CIs, p-values and FDR corrections for the full stratified analyses of the Irish cohorts**.**

| Gene | SNP | Allele | Ca/Co | Case | Control | Test | OR | 95%CI | P | FDR |
|---|---|---|---|---|---|---|---|---|---|---|
| ***GPX1*** | rs1050450 | C>T | 209/359 | Polyp/Adenoma/HGD | Control | Dominant | 1.03 | 0.72, 1.45 | 0.875 | 0.99 |
| | | | 209/359 | Polyp/Adenoma/HGD | Control | Recessive | 0.95 | 0.54, 1.65 | 0.850 | 0.99 |
| | | | 209/359 | Polyp/Adenoma/HGD | Control | Additive | 1.00 | 0.77, 1.29 | 0.977 | 1.00 |
| | | | 230/359 | Cancer | Control | Dominant | 0.85 | 0.61, 1.18 | 0.342 | 0.89 |
| | | | 230/359 | Cancer | Control | Recessive | 1.11 | 0.60, 1.72 | 0.951 | 1.00 |
| | | | 230/359 | Cancer | Control | Additive | 0.92 | 0.72, 1.18 | 0.499 | 0.89 |
| | | | 230/468 | Cancer | Control/Polyp | Dominant | 0.87 | 0.63, 1.18 | 0.368 | 0.89 |
| | | | 230/468 | Cancer | Control/Polyp | Recessive | 1.00 | 0.62, 1.64 | 0.994 | 1.00 |
| | | | 230/468 | Cancer | Control/Polyp | Additive | 1.20 | 0.81, 1.78 | 0.503 | 0.89 |
| | | | 355/468 | Adenoma/HGD/Cancer | Control/Polyp | Dominant | 0.94 | 0.71, 1.25 | 0.689 | 0.94 |
| | | | 355/468 | Adenoma/HGD/Cancer | Control/Polyp | Recessive | 0.94 | 0.59, 1.47 | 0.772 | 0.95 |
| | | | 355/468 | Adenoma/HGD/Cancer | Control/Polyp | Additive | 0.95 | 0.77, 1.18 | 0.664 | 0.94 |
| | | | 355/359 | Adenoma/HGD/Cancer | Control | Dominant | 0.93 | 0.14, 0.70 | 0.615 | 0.92 |
| | | | 355/359 | Adenoma/HGD/Cancer | Control | Recessive | 0.98 | 0.63, 1.54 | 0.944 | 1.00 |
| | | | 355/359 | Adenoma/HGD/Cancer | Control | Additive | 0.96 | 0.77, 1.18 | 0.684 | 0.94 |
| | | | 99/359 | Adenoma/HGD | Control | Dominant | 1.14 | 0.73, 1.78 | 0.562 | 0.91 |
| | | | 99/359 | Adenoma/HGD | Control | Recessive | 0.81 | 0.38, 1.72 | 0.579 | 0.91 |
| | | | 99/359 | Adenoma/HGD | Control | Additive | 1.03 | 0.74, 1.44 | 0.860 | 0.99 |
| | | | 99/468 | Adenoma/HGD | Control/Polyp | Dominant | 1.16 | 0.75, 1.80 | 0.506 | 0.89 |
| | | | 99/468 | Adenoma/HGD | Control/Polyp | Recessive | 0.79 | 0.38, 1.66 | 0.538 | 0.89 |
| | | | 99/468 | Adenoma/HGD | Control/Polyp | Additive | 1.04 | 0.75, 1.43 | 0.835 | 0.99 |
| ***GPX3*** | rs8177426 | A>G | 209/364 | Polyp/Adenoma/HGD | Control | Dominant | 0.93 | 0.64, 1.34 | 0.680 | 0.94 |
| | | | 209/364 | Polyp/Adenoma/HGD | Control | Recessive | 1.11 | 0.36, 3.43 | 0.860 | 0.99 |
| | | | 209/364 | Polyp/Adenoma/HGD | Control | Additive | 0.95 | 0.68, 1.32 | 0.749 | 0.94 |
| | | | 240/364 | Cancer | Control | Dominant | 1.18 | 0.84, 1.67 | 0.332 | 0.89 |
| | | | 240/364 | Cancer | Control | Recessive | 0.95 | 0.31, 2.93 | 0.924 | 0.99 |
| | | | 240/364 | Cancer | Control | Additive | 1.14 | 0.84, 1.56 | 0.394 | 0.89 |

| | | | | | | | | | | |
|---|---|---|---|---|---|---|---|---|---|---|
| | | | 240/471 | Cancer | Control/Polyp | Dominant | 1.16 | 0.85, 1.59 | 0.379 | 0.89 |
| | | | 240/471 | Cancer | Control/Polyp | Recessive | 0.81 | 0.28, 2.34 | 0.702 | 0.94 |
| | | | 240/471 | Cancer | Control/Polyp | Additive | 1.11 | 0.83, 1.48 | 0.497 | 0.89 |
| | | | 358/471 | Adenoma/HGD/Cancer | Control/Polyp | Dominant | 1.02 | 0.76, 1.37 | 0.891 | 0.99 |
| | | | 358/471 | Adenoma/HGD/Cancer | Control/Polyp | Recessive | 0.69 | 0.26, 1.86 | 0.461 | 0.89 |
| | | | 358/471 | Adenoma/HGD/Cancer | Control/Polyp | Additive | 0.99 | 0.76, 1.29 | 0.932 | 0.99 |
| | | | 358/364 | Adenoma/HGD/Cancer | Control | Dominant | 1.06 | 0.79, 1.42 | 0.700 | 0.94 |
| | | | 358/364 | Adenoma/HGD/Cancer | Control | Recessive | 1.02 | 0.39, 2.61 | 0.966 | 1.00 |
| | | | 358/364 | Adenoma/HGD/Cancer | Control | Additive | 1.05 | 0.81, 1.37 | 0.719 | 0.94 |
| | | | 99/364 | Adenoma/HGD | Control | Dominant | 0.75 | 0.46, 1.23 | 0.260 | 0.85 |
| | | | 99/364 | Adenoma/HGD | Control | Recessive | 0.45 | 0.06, 3.67 | 0.459 | 0.89 |
| | | | 99/364 | Adenoma/HGD | Control | Additive | 0.75 | 0.47, 1.19 | 0.220 | 0.83 |
| | | | 99/471 | Adenoma/HGD | Control/Polyp | Dominant | 0.74 | 0.45, 1.19 | 0.212 | 0.83 |
| | | | 99/471 | Adenoma/HGD | Control/Polyp | Recessive | 0.39 | 0.05, 3.04 | 0.369 | 0.89 |
| | | | 99/471 | Adenoma/HGD | Control/Polyp | Additive | 0.73 | 0.47, 1.14 | 0.166 | 0.80 |
| ***GPX3*** | rs8177447 | T>C | 206/363 | Polyp/Adenoma/HGD | Control | Dominant | 0.97 | 0.67, 1.39 | 0.862 | 0.99 |
| | | | 206/363 | Polyp/Adenoma/HGD | Control | Recessive | 1.09 | 0.36, 3.38 | 0.878 | 0.99 |
| | | | 206/363 | Polyp/Adenoma/HGD | Control | Additive | 0.98 | 0.71, 1.36 | 0.911 | 0.99 |
| | | | 238/363 | Cancer | Control | Dominant | 1.14 | 0.81, 1.61 | 0.457 | 0.89 |
| | | | 238/363 | Cancer | Control | Recessive | 0.95 | 0.31, 2.94 | 0.932 | 0.99 |
| | | | 238/363 | Cancer | Control | Additive | 1.11 | 0.81, 1.52 | 0.517 | 0.89 |
| | | | 238/472 | Cancer | Control/Polyp | Dominant | 1.10 | 0.79, 1.52 | 0.588 | 0.91 |
| | | | 238/472 | Cancer | Control/Polyp | Recessive | 0.82 | 0.29, 2.36 | 0.717 | 0.94 |
| | | | 238/472 | Cancer | Control/Polyp | Additive | 1.06 | 0.79, 1.42 | 0.704 | 0.94 |
| | | | 356/472 | Adenoma/HGD/Cancer | Control/Polyp | Dominant | 0.98 | 0.73, 1.32 | 0.882 | 0.99 |
| | | | 356/472 | Adenoma/HGD/Cancer | Control/Polyp | Recessive | 0.70 | 0.29, 1.87 | 0.471 | 0.89 |
| | | | 356/472 | Adenoma/HGD/Cancer | Control/Polyp | Additive | 0.96 | 0.73, 1.25 | 0.735 | 0.94 |
| | | | 356/363 | Adenoma/HGD/Cancer | Control | Dominant | 1.06 | 0.79, 1.42 | 0.711 | 0.94 |
| | | | 356/363 | Adenoma/HGD/Cancer | Control | Recessive | 1.02 | 0.39, 2.61 | 0.971 | 1.00 |
| | | | 356/363 | Adenoma/HGD/Cancer | Control | Additive | 1.05 | 0.80, 1.37 | 0.731 | 0.94 |

| | | | | | | | | | | |
|---|---|---|---|---|---|---|---|---|---|---|
| | | | 98/363 | Adenoma/HGD | Control | Dominant | 0.76 | 0.46, 1.25 | 0.277 | 0.86 |
| | | | 98/363 | Adenoma/HGD | Control | Recessive | 0.45 | 0.06, 3.67 | 0.458 | 0.89 |
| | | | 98/363 | Adenoma/HGD | Control | Additive | 0.76 | 0.48, 1.19 | 0.234 | 0.83 |
| | | | 98/472 | Adenoma/HGD | Control/Polyp | Dominant | 0.73 | 0.45, 1.19 | 0.202 | 0.83 |
| | | | 98/472 | Adenoma/HGD | Control/Polyp | Recessive | 0.39 | 0.05, 3.04 | 0.370 | 0.89 |
| | | | 98/472 | Adenoma/HGD | Control/Polyp | Additive | 0.73 | 0.46, 1.14 | 0.159 | 0.80 |
| ***GPX4*** | rs2074451 | T>G | 209/206 | Polyp/Adenoma/HGD | Control | Dominant | 1.00 | 0.69, 1.45 | 0.995 | 1.00 |
| | | | 209/206 | Polyp/Adenoma/HGD | Control | Recessive | 1.16 | 0.77, 1.75 | 0.477 | 0.89 |
| | | | 209/206 | Polyp/Adenoma/HGD | Control | Additive | 1.05 | 0.83, 1.33 | 0.688 | 0.94 |
| | | | 224/206 | Cancer | Control | Dominant | 1.08 | 0.75, 1.55 | 0.681 | 0.94 |
| | | | 224/206 | Cancer | Control | Recessive | 1.15 | 0.77, 1.72 | 0.493 | 0.89 |
| | | | 224/206 | Cancer | Control | Additive | 1.08 | 0.86, 1.36 | 0.512 | 0.89 |
| | | | 224/315 | Cancer | Control/Polyp | Dominant | 0.70 | 0.47, 1.03 | 0.073 | 0.72 |
| | | | 224/315 | Cancer | Control/Polyp | Recessive | 0.85 | 0.57, 1.26 | 0.408 | 0.89 |
| | | | 224/315 | Cancer | Control/Polyp | Additive | 0.82 | 0.65, 1.05 | 0.109 | 0.80 |
| | | | 355/315 | Adenoma/HGD/Cancer | Control/Polyp | Dominant | 0.70 | 0.49, 1.01 | 0.055 | 0.70 |
| | | | 355/315 | Adenoma/HGD/Cancer | Control/Polyp | Recessive | 0.91 | 0.64, 1.29 | 0.599 | 0.91 |
| | | | 355/315 | Adenoma/HGD/Cancer | Control/Polyp | Additive | 0.85 | 0.68, 1.05 | 0.130 | 0.80 |
| | | | 355/206 | Adenoma/HGD/Cancer | Control | Dominant | 1.04 | 0.77, 1.41 | 0.803 | 0.97 |
| | | | 355/206 | Adenoma/HGD/Cancer | Control | Recessive | 1.16 | 0.82, 1.62 | 0.405 | 0.89 |
| | | | 355/206 | Adenoma/HGD/Cancer | Control | Additive | 1.07 | 0.88, 1.29 | 0.525 | 0.89 |
| | | | 98/206 | Adenoma/HGD | Control | Dominant | 0.92 | 0.57, 1.48 | 0.735 | 0.94 |
| | | | 98/206 | Adenoma/HGD | Control | Recessive | 1.32 | 0.78, 2.21 | 0.299 | 0.88 |
| | | | 98/206 | Adenoma/HGD | Control | Additive | 1.06 | 0.78, 1.44 | 0.706 | 0.94 |
| | | | 98/315 | Adenoma/HGD | Control/Polyp | Dominant | 0.94 | 0.56, 1.56 | 0.814 | 0.97 |
| | | | 98/315 | Adenoma/HGD | Control/Polyp | Recessive | 1.40 | 0.84, 2.34 | 0.198 | 0.83 |
| | | | 98/315 | Adenoma/HGD | Control/Polyp | Additive | 0.16 | 0.80, 1.53 | 0.530 | 0.89 |
| ***GPX4*** | rs713041 | T>C | 209/284 | Polyp/Adenoma/HGD | Control | Dominant | 0.68 | 0.45, 1.01 | 0.059 | 0.71 |
| | | | 209/284 | Polyp/Adenoma/HGD | Control | Recessive | 1.13 | 0.65, 1.94 | 0.674 | 0.94 |
| | | | 209/284 | Polyp/Adenoma/HGD | Control | Additive | 0.85 | 0.64, 1.13 | 0.261 | 0.85 |

| | | | | | | | | | | |
|---|---|---|---|---|---|---|---|---|---|---|
| | | | 225/284 | Cancer | Control | Dominant | 0.76 | 0.52, 1.13 | 0.177 | 0.80 |
| | | | 225/284 | Cancer | Control | Recessive | 0.95 | 0.55, 1.64 | 0.864 | 0.99 |
| | | | 225/284 | Cancer | Control | Additive | 0.86 | 0.65, 1.14 | 0.288 | 0.87 |
| | | | 225/448 | Cancer | Control/Polyp | Dominant | 1.06 | 0.75, 1.50 | 0.741 | 0.94 |
| | | | 225/448 | Cancer | Control/Polyp | Recessive | 1.14 | 0.78, 1.67 | 0.491 | 0.89 |
| | | | 225/448 | Cancer | Control/Polyp | Additive | 1.07 | 0.86, 1.34 | 0.544 | 0.89 |
| | | | 355/448 | Adenoma/HGD/Cancer | Control/Polyp | Dominant | 1.01 | 0.74, 1.37 | 0.952 | 1.00 |
| | | | 355/448 | Adenoma/HGD/Cancer | Control/Polyp | Recessive | 1.19 | 0.85, 1.67 | 0.310 | 0.89 |
| | | | 355/448 | Adenoma/HGD/Cancer | Control/Polyp | Additive | 1.06 | 0.87, 1.29 | 0.530 | 0.89 |
| | | | 355/284 | Adenoma/HGD/Cancer | Control | Dominant | 0.72 | 0.51, 1.02 | 0.066 | 0.72 |
| | | | 355/284 | Adenoma/HGD/Cancer | Control | Recessive | 1.03 | 0.64, 1.66 | 0.896 | 0.99 |
| | | | 355/284 | Adenoma/HGD/Cancer | Control | Additive | 0.86 | 0.67, 1.09 | 0.213 | 0.83 |
| | | | 98/284 | Adenoma/HGD | Control | Dominant | 0.71 | 0.48, 1.17 | 0.181 | 0.80 |
| | | | 98/284 | Adenoma/HGD | Control | Recessive | 0.81 | 0.39, 1.70 | 0.582 | 0.91 |
| | | | 98/284 | Adenoma/HGD | Control | Additive | 0.79 | 0.54, 1.14 | 0.210 | 0.83 |
| | | | 98/448 | Adenoma/HGD | Control/Polyp | Dominant | 0.91 | 0.57, 1.44 | 0.672 | 0.94 |
| | | | 98/448 | Adenoma/HGD | Control/Polyp | Recessive | 1.31 | 0.79, 2.16 | 0.296 | 0.88 |
| | | | 98/448 | Adenoma/HGD | Control/Polyp | Additive | 1.05 | 0.78, 1.41 | 0.740 | 0.94 |
| ***GPX5*** | rs445870 | A>G | 209/215 | Polyp/Adenoma/HGD | Control | Dominant | 0.92 | 0.61, 1.37 | 0.668 | 0.94 |
| | | | 209/215 | Polyp/Adenoma/HGD | Control | Recessive | 2.12 | 0.63, 7.16 | 0.225 | 0.83 |
| | | | 209/215 | Polyp/Adenoma/HGD | Control | Additive | 1.00 | 0.70, 1.43 | 0.997 | 1.00 |
| | | | 236/215 | Cancer | Control | Dominant | 0.87 | 0.59, 1.29 | 0.489 | 0.89 |
| | | | 236/215 | Cancer | Control | Recessive | 2.25 | 0.69, 7.29 | 0.176 | 0.80 |
| | | | 236/215 | Cancer | Control | Additive | 0.97 | 0.69, 1.36 | 0.870 | 0.99 |
| | | | 236/324 | Cancer | Control/Polyp | Dominant | 1.19 | 0.85, 1.66 | 0.317 | 0.89 |
| | | | 236/324 | Cancer | Control/Polyp | Recessive | 0.66 | 0.37, 1.18 | 0.158 | 0.80 |
| | | | 236/324 | Cancer | Control/Polyp | Additive | 1.01 | 0.79, 1.30 | 0.916 | 0.99 |
| | | | 355/324 | Adenoma/HGD/Cancer | Control/Polyp | Dominant | 1.12 | 0.83, 1.53 | 0.456 | 0.89 |
| | | | 355/324 | Adenoma/HGD/Cancer | Control/Polyp | Recessive | 0.69 | 0.41, 1.16 | 0.159 | 0.80 |
| | | | 355/324 | Adenoma/HGD/Cancer | Control/Polyp | Additive | 0.99 | 0.79, 1.25 | 0.937 | 1.00 |

| | | | | | | | | | | |
|---|---|---|---|---|---|---|---|---|---|---|
| | | | 355/215 | Adenoma/HGD/Cancer | Control | Dominant | 0.89 | 0.63, 1.26 | 0.512 | 0.89 |
| | | | 355/215 | Adenoma/HGD/Cancer | Control | Recessive | 2.19 | 0.73, 6.56 | 0.160 | 0.80 |
| | | | 355/215 | Adenoma/HGD/Cancer | Control | Additive | 0.99 | 0.73, 1.33 | 0.923 | 0.99 |
| | | | 98/215 | Adenoma/HGD | Control | Dominant | 1.11 | 0.68, 1.82 | 0.687 | 0.94 |
| | | | 98/215 | Adenoma/HGD | Control | Recessive | 2.27 | 0.56, 9.28 | 0.253 | 0.85 |
| | | | 98/215 | Adenoma/HGD | Control | Additive | 1.17 | 0.76, 1.82 | 0.481 | 0.89 |
| | | | 98/324 | Adenoma/HGD | Control/Polyp | Dominant | 0.98 | 0.62, 1.55 | 0.943 | 1.00 |
| | | | 98/324 | Adenoma/HGD | Control/Polyp | Recessive | 0.77 | 0.36, 1.65 | 0.503 | 0.89 |
| | | | 98/324 | Adenoma/HGD | Control/Polyp | Additive | 0.94 | 0.67, 1.31 | 0.716 | 0.94 |
| ***SELENOF*** | rs5859 | C>T | 209/368 | Polyp/Adenoma/HGD | Control | Dominant | 1.29 | 0.91, 1.83 | 0.153 | 0.80 |
| | | | 209/368 | Polyp/Adenoma/HGD | Control | Recessive | 0.82 | 0.78, 4.28 | 0.169 | 0.80 |
| | | | 209/368 | Polyp/Adenoma/HGD | Control | Additive | 1.29 | 0.96, 1.74 | 0.090 | 0.75 |
| | | | 236/368 | Cancer | Control | Dominant | 1.54 | 1.10, 2.15 | **0.012** | 0.50 |
| | | | 236/368 | Cancer | Control | Recessive | 2.05 | 0.91, 4.59 | 0.082 | 0.75 |
| | | | 236/368 | Cancer | Control | Additive | 1.49 | 1.12, 1.98 | **0.006** | 0.50 |
| | | | 236/477 | Cancer | Control/Polyp | Dominant | 1.47 | 1.07, 2.02 | **0.017** | 0.50 |
| | | | 236/477 | Cancer | Control/Polyp | Recessive | 1.82 | 0.87, 3.80 | 0.111 | 0.80 |
| | | | 236/477 | Cancer | Control/Polyp | Additive | 1.42 | 1.09, 1.86 | **0.010** | 0.50 |
| | | | 334/477 | Adenoma/HGD/Cancer | Control/Polyp | Dominant | 1.43 | 1.07, 1.90 | **0.014** | 0.50 |
| | | | 334/477 | Adenoma/HGD/Cancer | Control/Polyp | Recessive | 1.84 | 0.94, 3.59 | **0.008** | 0.50 |
| | | | 334/477 | Adenoma/HGD/Cancer | Control/Polyp | Additive | 1.39 | 1.10, 1.77 | **0.007** | 0.50 |
| | | | 334/368 | Adenoma/HGD/Cancer | Control | Dominant | 1.42 | 1.07, 1.88 | **0.016** | 0.50 |
| | | | 334/368 | Adenoma/HGD/Cancer | Control | Recessive | 1.94 | 0.37, 0.95 | 0.072 | 0.72 |
| | | | 334/368 | Adenoma/HGD/Cancer | Control | Additive | 1.39 | 1.09, 1.78 | **0.008** | 0.50 |
| | | | 99/368 | Adenoma/HGD | Control | Dominant | 1.39 | 0.88, 2.19 | 0.155 | 0.80 |
| | | | 99/368 | Adenoma/HGD | Control | Recessive | 2.12 | 0.76, 5.87 | 0.150 | 0.80 |
| | | | 99/368 | Adenoma/HGD | Control | Additive | 1.40 | 0.95, 2.04 | 0.087 | 0.75 |
| | | | 99/477 | Adenoma/HGD | Control/Polyp | Dominant | 1.33 | 0.86, 2.06 | 0.206 | 0.83 |
| | | | 99/477 | Adenoma/HGD | Control/Polyp | Recessive | 1.88 | 0.72, 4.93 | 0.200 | 0.83 |
| | | | 99/477 | Adenoma/HGD | Control/Polyp | Additive | 1.33 | 0.92, 1.93 | 0.127 | 0.80 |

| | | | | | | | | | | |
|---|---|---|---|---|---|---|---|---|---|---|
| ***SELENOM*** | rs11705137 | T>C | 209/209 | Polyp/Adenoma/HGD | Control | Dominant | 1.31 | 0.93, 1.85 | 0.127 | 0.80 |
| | | | 209/209 | Polyp/Adenoma/HGD | Control | Recessive | 1.25 | 0.64, 2.42 | 0.510 | 0.89 |
| | | | 209/209 | Polyp/Adenoma/HGD | Control | Additive | 1.23 | 0.94, 1.61 | 0.140 | 0.80 |
| | | | 225/209 | Cancer | Control | Dominant | 1.30 | 0.93, 1.81 | 0.123 | 0.80 |
| | | | 225/209 | Cancer | Control | Recessive | 1.10 | 0.57, 2.12 | 0.789 | 0.95 |
| | | | 225/209 | Cancer | Control | Additive | 1.20 | 0.92, 1.56 | 0.179 | 0.80 |
| | | | 225/316 | Cancer | Control/Polyp | Dominant | 1.33 | 0.88, 1.99 | 0.174 | 0.80 |
| | | | 225/316 | Cancer | Control/Polyp | Recessive | 0.99 | 0.66, 1.48 | 0.962 | 1.00 |
| | | | 225/316 | Cancer | Control/Polyp | Additive | 1.11 | 0.87, 1.42 | 0.417 | 0.89 |
| | | | 352/316 | Adenoma/HGD/Cancer | Control/Polyp | Dominant | 1.13 | 0.79, 1.62 | 0.507 | 0.89 |
| | | | 352/316 | Adenoma/HGD/Cancer | Control/Polyp | Recessive | 1.10 | 0.77, 1.58 | 0.596 | 0.91 |
| | | | 352/316 | Adenoma/HGD/Cancer | Control/Polyp | Additive | 1.09 | 0.87, 1.36 | 0.460 | 0.89 |
| | | | 352/209 | Adenoma/HGD/Cancer | Control | Dominant | 1.30 | 0.98, 1.73 | 0.065 | 0.72 |
| | | | 352/209 | Adenoma/HGD/Cancer | Control | Recessive | 1.17 | 0.67, 2.03 | 0.586 | 0.91 |
| | | | 352/209 | Adenoma/HGD/Cancer | Control | Additive | 1.21 | 0.97, 1.52 | 0.091 | 0.75 |
| | | | 99/209 | Adenoma/HGD | Control | Dominant | 0.99 | 0.63, 1.57 | 0.976 | 1.00 |
| | | | 99/209 | Adenoma/HGD | Control | Recessive | 1.16 | 0.48, 2.79 | 0.740 | 0.94 |
| | | | 99/209 | Adenoma/HGD | Control | Additive | 1.02 | 0.71, 1.47 | 0.913 | 0.99 |
| | | | 99/316 | Adenoma/HGD | Control/Polyp | Dominant | 0.81 | 0.49, 1.34 | 0.411 | 0.89 |
| | | | 99/316 | Adenoma/HGD | Control/Polyp | Recessive | 1.39 | 0.84, 2.31 | 0.202 | 0.83 |
| | | | 99/316 | Adenoma/HGD | Control/Polyp | Additive | 1.04 | 0.76, 1.43 | 0.790 | 0.95 |
| ***SELENON*** | rs11247710 | G>C | 209/219 | Polyp/Adenoma/HGD | Control | Dominant | 1.07 | 0.71, 1.61 | 0.753 | 0.94 |
| | | | 209/219 | Polyp/Adenoma/HGD | Control | Recessive | 1.09 | 0.68, 1.74 | 0.727 | 0.94 |
| | | | 209/219 | Polyp/Adenoma/HGD | Control | Additive | 1.06 | 0.81, 1.37 | 0.690 | 0.94 |
| | | | 215/219 | Cancer | Control | Dominant | 1.16 | 0.77, 1.74 | 0.476 | 0.89 |
| | | | 215/219 | Cancer | Control | Recessive | 1.08 | 0.68, 1.71 | 0.749 | 0.94 |
| | | | 215/219 | Cancer | Control | Additive | 1.09 | 0.84, 1.41 | 0.521 | 0.89 |
| | | | 215/320 | Cancer | Control/Polyp | Dominant | 1.19 | 0.82, 1.72 | 0.359 | 0.89 |
| | | | 215/320 | Cancer | Control/Polyp | Recessive | 1.12 | 0.74, 1.71 | 0.590 | 0.91 |
| | | | 215/320 | Cancer | Control/Polyp | Additive | 0.92 | 0.73, 1.17 | 0.369 | 0.89 |

| | | | | | | | | | | |
|---|---|---|---|---|---|---|---|---|---|---|
| | | | 356/320 | Adenoma/HGD/Cancer | Control/Polyp | Dominant | 1.22 | 0.87, 1.73 | 0.245 | 0.84 |
| | | | 356/320 | Adenoma/HGD/Cancer | Control/Polyp | Recessive | 1.20 | 0.82, 1.76 | 0.342 | 0.89 |
| | | | 356/320 | Adenoma/HGD/Cancer | Control/Polyp | Additive | 1.15 | 0.93, 1.43 | 0.190 | 0.82 |
| | | | 356/219 | Adenoma/HGD/Cancer | Control | Dominant | 1.11 | 0.78, 1.58 | 0.548 | 0.90 |
| | | | 356/219 | Adenoma/HGD/Cancer | Control | Recessive | 1.08 | 0.72, 1.62 | 0.701 | 0.94 |
| | | | 356/219 | Adenoma/HGD/Cancer | Control | Additive | 1.07 | 0.86, 1.35 | 0.545 | 0.89 |
| | | | 99/219 | Adenoma/HGD | Control | Dominant | 1.26 | 0.75, 2.12 | 0.389 | 0.89 |
| | | | 99/219 | Adenoma/HGD | Control | Recessive | 1.33 | 0.76, 2.34 | 0.316 | 0.89 |
| | | | 99/219 | Adenoma/HGD | Control | Additive | 1.21 | 0.87, 1.68 | 0.263 | 0.85 |
| | | | 99/320 | Adenoma/HGD | Control/Polyp | Dominant | 1.29 | 0.79, 2.12 | 0.313 | 0.89 |
| | | | 99/320 | Adenoma/HGD | Control/Polyp | Additive | 1.24 | 0.91, 1.65 | 0.182 | 0.80 |
| ***SELENON*** | rs4659382 | C>G | 206/214 | Polyp/Adenoma/HGD | Control | Dominant | 1.43 | 0.97, 2.11 | 0.070 | 0.72 |
| | | | 206/214 | Polyp/Adenoma/HGD | Control | Recessive | 3.52 | 1.27, 9.79 | **0.016** | 0.50 |
| | | | 206/214 | Polyp/Adenoma/HGD | Control | Additive | 1.51 | 1.08, 2.07 | **0.015** | 0.50 |
| | | | 236/214 | Cancer | Control | Dominant | 1.17 | 0.79, 1.70 | 0.427 | 0.89 |
| | | | 236/214 | Cancer | Control | Recessive | 2.64 | 0.93, 7.45 | 0.067 | 0.72 |
| | | | 236/214 | Cancer | Control | Additive | 1.25 | 0.90, 1.73 | 0.181 | 0.80 |
| | | | 236/323 | Cancer | Control/Polyp | Dominant | 1.07 | 0.76, 1.50 | 0.706 | 0.94 |
| | | | 236/323 | Cancer | Control/Polyp | Recessive | 1.39 | 0.65, 2.98 | 0.394 | 0.89 |
| | | | 236/323 | Cancer | Control/Polyp | Additive | 1.10 | 0.83, 1.45 | 0.529 | 0.89 |
| | | | 333/323 | Adenoma/HGD/Cancer | Control/Polyp | Dominant | 1.17 | 0.86, 1.60 | 0.311 | 0.89 |
| | | | 333/323 | Adenoma/HGD/Cancer | Control/Polyp | Recessive | 1.49 | 0.74, 2.98 | 0.264 | 0.85 |
| | | | 333/323 | Adenoma/HGD/Cancer | Control/Polyp | Additive | 1.18 | 0.91, 1.53 | 0.207 | 0.83 |
| | | | 333/214 | Adenoma/HGD/Cancer | Control | Dominant | 1.28 | 0.92, 1.79 | 0.142 | 0.80 |
| | | | 333/214 | Adenoma/HGD/Cancer | Control | Recessive | 3.04 | 1.16, 7.96 | **0.023** | 0.57 |
| | | | 333/214 | Adenoma/HGD/Cancer | Control | Additive | 1.36 | 1.02, 1.80 | **0.035** | 0.68 |
| | | | 97/214 | Adenoma/HGD | Control | Dominant | 1.61 | 0.99, 2.61 | 0.055 | 0.70 |
| | | | 97/214 | Adenoma/HGD | Control | Recessive | 3.25 | 1.01, 10.52 | **0.049** | 0.69 |
| | | | 97/214 | Adenoma/HGD | Control | Additive | 1.64 | 1.09, 2.48 | **0.019** | 0.50 |
| | | | 97/323 | Adenoma/HGD | Control/Polyp | Dominant | 1.47 | 0.94, 2.32 | 0.096 | 0.75 |

| | | | | | | | | | | |
|---|---|---|---|---|---|---|---|---|---|---|
| | | | 97/323 | Adenoma/HGD | Control/Polyp | Recessive | 1.72 | 0.48, 0.67 | 0.258 | 0.85 |
| | | | 97/323 | Adenoma/HGD | Control/Polyp | Additive | 1.41 | 0.97, 2.04 | 0.071 | 0.72 |
| ***SELENOP*** | rs2972994 | G>A | 208/363 | Polyp/Adenoma/HGD | Control | Dominant | 1.10 | 0.75, 1.59 | 0.630 | 0.92 |
| | | | 208/363 | Polyp/Adenoma/HGD | Control | Recessive | 1.08 | 0.72, 1.62 | 0.710 | 0.94 |
| | | | 208/363 | Polyp/Adenoma/HGD | Control | Additive | 1.06 | 0.84, 1.35 | 0.603 | 0.91 |
| | | | 240/363 | Cancer | Control | Dominant | 1.44 | 0.99, 2.08 | 0.055 | 0.70 |
| | | | 240/363 | Cancer | Control | Recessive | 0.14 | 0.77, 1.67 | 0.518 | 0.89 |
| | | | 240/363 | Cancer | Control | Additive | 1.20 | 0.96, 1.51 | 0.112 | 0.80 |
| | | | 240/471 | Cancer | Control/Polyp | Dominant | 1.44 | 1.01, 2.05 | **0.044** | 0.69 |
| | | | 240/471 | Cancer | Control/Polyp | Recessive | 1.18 | 0.82, 1.70 | 0.378 | 0.89 |
| | | | 240/471 | Cancer | Control/Polyp | Additive | 1.22 | 0.98, 1.52 | 0.073 | 0.72 |
| | | | 308/471 | Adenoma/HGD/Cancer | Control/Polyp | Dominant | 1.37 | 1.00, 1.87 | **0.049** | 0.69 |
| | | | 308/471 | Adenoma/HGD/Cancer | Control/Polyp | Recessive | 1.25 | 0.89, 1.73 | 0.184 | 0.80 |
| | | | 308/471 | Adenoma/HGD/Cancer | Control/Polyp | Additive | 1.22 | 1.01, 1.49 | **0.044** | 0.69 |
| | | | 308/363 | Adenoma/HGD/Cancer | Control | Dominant | 1.26 | 0.93, 1.71 | 0.135 | 0.80 |
| | | | 308/363 | Adenoma/HGD/Cancer | Control | Recessive | 1.11 | 0.79, 1.54 | 0.537 | 0.89 |
| | | | 308/363 | Adenoma/HGD/Cancer | Control | Additive | 0.14 | 0.93, 1.38 | 0.193 | 0.82 |
| | | | 98/363 | Adenoma/HGD | Control | Dominant | 1.22 | 0.74, 2.00 | 0.429 | 0.89 |
| | | | 98/363 | Adenoma/HGD | Control | Recessive | 1.37 | 0.83, 2.27 | 0.216 | 0.83 |
| | | | 98/363 | Adenoma/HGD | Control | Additive | 1.21 | 0.89, 1.64 | 0.225 | 0.83 |
| | | | 98/471 | Adenoma/HGD | Control/Polyp | Dominant | 1.22 | 0.76, 1.98 | 0.416 | 0.89 |
| | | | 98/471 | Adenoma/HGD | Control/Polyp | Recessive | 1.43 | 0.88, 2.33 | 0.155 | 0.80 |
| | | | 98/471 | Adenoma/HGD | Control/Polyp | Additive | 1.23 | 0.59, 1.21 | 0.180 | 0.80 |
| ***SELENOP*** | rs7579 | T>C | 209/364 | Polyp/Adenoma/HGD | Control | Dominant | 1.18 | 0.83, 1.66 | 0.355 | 0.89 |
| | | | 209/364 | Polyp/Adenoma/HGD | Control | Recessive | 1.74 | 0.90, 3.34 | 0.097 | 0.75 |
| | | | 209/364 | Polyp/Adenoma/HGD | Control | Additive | 1.22 | 0.93, 1.59 | 0.156 | 0.80 |
| | | | 233/364 | Cancer | Control | Dominant | 1.19 | 0.86, 1.66 | 0.297 | 0.88 |
| | | | 233/364 | Cancer | Control | Recessive | 1.35 | 0.69, 2.64 | 0.375 | 0.89 |
| | | | 233/364 | Cancer | Control | Additive | 1.18 | 0.90, 1.54 | 0.231 | 0.83 |
| | | | 233/473 | Cancer | Control/Polyp | Dominant | 1.08 | 0.79, 1.48 | 0.622 | 0.92 |

| | | | | | | | | | | |
|---|---|---|---|---|---|---|---|---|---|---|
| | | | 233/473 | Cancer | Control/Polyp | Recessive | 1.05 | 0.57, 1.93 | 0.876 | 0.99 |
| | | | 233/473 | Cancer | Control/Polyp | Additive | 1.06 | 0.83, 1.36 | 0.648 | 0.94 |
| | | | 356/473 | Adenoma/HGD/Cancer | Control/Polyp | Dominant | 0.99 | 0.75, 1.31 | 0.949 | 1.00 |
| | | | 356/473 | Adenoma/HGD/Cancer | Control/Polyp | Recessive | 1.00 | 0.57, 1.73 | 0.988 | 1.00 |
| | | | 356/473 | Adenoma/HGD/Cancer | Control/Polyp | Additive | 0.99 | 0.79, 1.24 | 0.954 | 1.00 |
| | | | 356/364 | Adenoma/HGD/Cancer | Control | Dominant | 1.19 | 0.89, 1.57 | 0.235 | 0.83 |
| | | | 356/364 | Adenoma/HGD/Cancer | Control | Recessive | 1.53 | 0.87, 2.69 | 0.139 | 0.80 |
| | | | 356/364 | Adenoma/HGD/Cancer | Control | Additive | 1.20 | 0.96, 1.49 | 0.120 | 0.80 |
| | | | 99/364 | Adenoma/HGD | Control | Dominant | 0.88 | 0.56, 1.39 | 0.586 | 0.91 |
| | | | 99/364 | Adenoma/HGD | Control | Recessive | 1.12 | 0.44, 2.87 | 0.811 | 0.97 |
| | | | 99/364 | Adenoma/HGD | Control | Additive | 0.93 | 0.64, 1.34 | 0.722 | 0.94 |
| | | | 99/473 | Adenoma/HGD | Control/Polyp | Dominant | 0.80 | 0.51, 1.25 | 0.324 | 0.89 |
| | | | 99/473 | Adenoma/HGD | Control/Polyp | Recessive | 0.87 | 0.35, 2.14 | 0.760 | 0.94 |
| | | | 99/473 | Adenoma/HGD | Control/Polyp | Additive | 0.85 | 0.59, 1.21 | 0.361 | 0.89 |
| ***SELENOS*** | rs34713741 | C>T | 209/362 | Polyp/Adenoma/HGD | Control | Dominant | 0.85 | 0.60, 1.19 | 0.350 | 0.89 |
| | | | 209/362 | Polyp/Adenoma/HGD | Control | Recessive | 1.25 | 0.62, 2.54 | 0.530 | 0.89 |
| | | | 209/362 | Polyp/Adenoma/HGD | Control | Additive | 0.93 | 0.69, 1.23 | 0.601 | 0.91 |
| | | | 235/362 | Cancer | Control | Dominant | 0.74 | 0.53, 1.04 | 0.080 | 0.75 |
| | | | 235/362 | Cancer | Control | Recessive | 1.77 | 0.94, 3.31 | 0.076 | 0.73 |
| | | | 235/362 | Cancer | Control | Additive | 0.91 | 0.70, 1.19 | 0.505 | 0.89 |
| | | | 235/472 | Cancer | Control/Polyp | Dominant | 0.73 | 0.53, 0.99 | **0.049** | 0.69 |
| | | | 235/472 | Cancer | Control/Polyp | Recessive | 1.58 | 0.88, 2.81 | 0.122 | 0.80 |
| | | | 235/472 | Cancer | Control/Polyp | Additive | 0.89 | 0.69, 1.14 | 0.352 | 0.89 |
| | | | 365/472 | Adenoma/HGD/Cancer | Control/Polyp | Dominant | 0.69 | 0.52, 0.92 | **0.011** | 0.50 |
| | | | 365/472 | Adenoma/HGD/Cancer | Control/Polyp | Recessive | 1.36 | 0.79, 2.35 | 0.267 | 0.85 |
| | | | 365/472 | Adenoma/HGD/Cancer | Control/Polyp | Additive | 0.83 | 0.66, 1.05 | 0.110 | 0.80 |
| | | | 365/362 | Adenoma/HGD/Cancer | Control | Dominant | 0.79 | 0.59, 1.05 | 0.101 | 0.77 |
| | | | 365/362 | Adenoma/HGD/Cancer | Control | Recessive | 1.52 | 0.87, 2.68 | 0.144 | 0.80 |
| | | | 365/362 | Adenoma/HGD/Cancer | Control | Additive | 0.92 | 0.74, 1.15 | 0.476 | 0.89 |
| | | | 95/362 | Adenoma/HGD | Control | Dominant | 0.62 | 0.39, 0.99 | **0.046** | 0.69 |

| | | | | | | | | | | |
|---|---|---|---|---|---|---|---|---|---|---|
| | | | 95/362 | Adenoma/HGD | Control | Recessive | 0.95 | 0.35, 2.60 | 0.921 | 0.99 |
| | | | 95/362 | Adenoma/HGD | Control | Additive | 0.71 | 0.48, 1.05 | 0.088 | 0.75 |
| | | | 95/472 | Adenoma/HGD | Control/Polyp | Dominant | 0.61 | 0.39, 0.94 | **0.032** | 0.68 |
| | | | 95/472 | Adenoma/HGD | Control/Polyp | Recessive | 0.85 | 0.32, 2.25 | 0.742 | 0.94 |
| | | | 95/472 | Adenoma/HGD | Control/Polyp | Additive | 0.69 | 0.47, 1.01 | 0.058 | 0.71 |
| ***SEPSH1*** | rs2275129 | C>G | 201/216 | Polyp/Adenoma/HGD | Control | Dominant | 1.03 | 0.66, 1.59 | 0.897 | 0.99 |
| | | | 201/216 | Polyp/Adenoma/HGD | Control | Recessive | 1.62 | 1.03, 2.55 | **0.034** | 0.68 |
| | | | 201/216 | Polyp/Adenoma/HGD | Control | Additive | 1.21 | 0.92, 1.59 | 0.171 | 0.80 |
| | | | 207/216 | Cancer | Control | Dominant | 0.90 | 0.58, 1.38 | 0.628 | 0.92 |
| | | | 207/216 | Cancer | Control | Recessive | 1.41 | 0.89, 2.22 | 0.133 | 0.80 |
| | | | 207/216 | Cancer | Control | Additive | 1.09 | 0.82, 1.42 | 0.552 | 0.90 |
| | | | 207/323 | Cancer | Control/Polyp | Dominant | 0.95 | 0.64, 1.39 | 0.785 | 0.95 |
| | | | 207/323 | Cancer | Control/Polyp | Recessive | 1.28 | 0.86, 1.93 | 0.227 | 0.83 |
| | | | 207/323 | Cancer | Control/Polyp | Additive | 1.07 | 0.84, 1.37 | 0.582 | 0.91 |
| | | | 301/323 | Adenoma/HGD/Cancer | Control/Polyp | Dominant | 1.05 | 0.74, 1.51 | 0.773 | 0.95 |
| | | | 301/323 | Adenoma/HGD/Cancer | Control/Polyp | Recessive | 1.44 | 1.01, 2.07 | **0.047** | 0.69 |
| | | | 301/323 | Adenoma/HGD/Cancer | Control/Polyp | Additive | 1.17 | 0.94, 1.45 | 0.160 | 0.80 |
| | | | 301/216 | Adenoma/HGD/Cancer | Control | Dominant | 0.96 | 0.66, 1.39 | 0.832 | 0.99 |
| | | | 301/216 | Adenoma/HGD/Cancer | Control | Recessive | 1.52 | 1.02, 2.25 | **0.039** | 0.69 |
| | | | 301/216 | Adenoma/HGD/Cancer | Control | Additive | 1.14 | 0.91, 1.44 | 0.263 | 0.85 |
| | | | 94/216 | Adenoma/HGD | Control | Dominant | 1.30 | 0.73, 2.31 | 0.383 | 0.89 |
| | | | 94/216 | Adenoma/HGD | Control | Recessive | 2.02 | 1.18, 3.46 | **0.011** | 0.50 |
| | | | 94/216 | Adenoma/HGD | Control | Additive | 1.46 | 1.03, 2.08 | **0.035** | 0.68 |
| | | | 94/323 | Adenoma/HGD | Control/Polyp | Dominant | 1.36 | 0.79, 2.37 | 0.270 | 0.85 |
| | | | 94/323 | Adenoma/HGD | Control/Polyp | Recessive | 1.83 | 1.11, 3.02 | **0.018** | 0.50 |
| | | | 94/323 | Adenoma/HGD | Control/Polyp | Additive | 1.43 | 1.03, 1.98 | **0.034** | 0.68 |
| *SOD2* | rs4880 | T>C | 209/366 | Polyp/Adenoma/HGD | Control | Dominant | 1.06 | 0.72, 1.57 | 0.752 | 0.94 |
| | | | 209/366 | Polyp/Adenoma/HGD | Control | Recessive | 0.90 | 0.60, 1.34 | 0.599 | 0.91 |
| | | | 209/366 | Polyp/Adenoma/HGD | Control | Additive | 0.99 | 0.78, 1.25 | 0.904 | 0.99 |
| | | | 236/366 | Cancer | Control | Dominant | 1.22 | 0.84, 1.78 | 0.304 | 0.89 |

| | | | | | | | | | | |
|---|---|---|---|---|---|---|---|---|---|---|
| | | | 236/366 | Cancer | Control | Recessive | 0.98 | 0.67, 1.43 | 0.910 | 0.99 |
| | | | 236/366 | Cancer | Control | Additive | 1.07 | 0.85, 1.34 | 0.575 | 0.91 |
| | | | 236/476 | Cancer | Control/Polyp | Recessive | 0.99 | 0.69, 1.42 | 0.952 | 1.00 |
| | | | 236/476 | Cancer | Control/Polyp | Additive | 1.06 | 0.85, 1.32 | 0.602 | 0.91 |
| | | | 355/476 | Adenoma/HGD/Cancer | Control/Polyp | Dominant | 1.11 | 0.80, 1.53 | 0.522 | 0.89 |
| | | | 355/476 | Adenoma/HGD/Cancer | Control/Polyp | Recessive | 0.95 | 0.68, 1.31 | 0.738 | 0.94 |
| | | | 355/476 | Adenoma/HGD/Cancer | Control/Polyp | Additive | 1.02 | 0.84, 1.24 | 0.849 | 0.99 |
| | | | 355/366 | Adenoma/HGD/Cancer | Control | Dominant | 1.14 | 0.83, 1.57 | 0.407 | 0.89 |
| | | | 355/366 | Adenoma/HGD/Cancer | Control | Recessive | 0.94 | 0.68, 1.29 | 0.708 | 0.94 |
| | | | 355/366 | Adenoma/HGD/Cancer | Control | Additive | 1.03 | 0.85, 1.25 | 0.778 | 0.95 |
| | | | 99/366 | Adenoma/HGD | Control | Dominant | 0.99 | 0.60, 1.63 | 0.965 | 1.00 |
| | | | 99/366 | Adenoma/HGD | Control | Recessive | 0.84 | 0.49, 1.42 | 0.515 | 0.89 |
| | | | 99/366 | Adenoma/HGD | Control | Additive | 0.94 | 0.69, 1.28 | 0.676 | 0.94 |
| | | | 99/476 | Adenoma/HGD | Control/Polyp | Dominant | 0.96 | 0.58, 1.56 | 0.870 | 0.99 |
| | | | 99/476 | Adenoma/HGD | Control/Polyp | Recessive | 0.85 | 0.51, 1.42 | 0.531 | 0.89 |
| | | | 99/476 | Adenoma/HGD | Control/Polyp | Additive | 0.93 | 0.69, 1.26 | 0.631 | 0.92 |
| *TXNRD1* | rs11111979 | C>G | 209/214 | Polyp/Adenoma/HGD | Control | Dominant | 1.13 | 0.74, 1.71 | 0.573 | 0.91 |
| | | | 209/214 | Polyp/Adenoma/HGD | Control | Recessive | 0.98 | 0.63, 1.53 | 0.924 | 0.99 |
| | | | 209/214 | Polyp/Adenoma/HGD | Control | Additive | 1.04 | 0.80, 1.35 | 0.766 | 0.95 |
| | | | 240/214 | Cancer | Control | Dominant | 1.15 | 0.77, 1.73 | 0.489 | 0.89 |
| | | | 240/214 | Cancer | Control | Recessive | 1.16 | 0.76, 1.77 | 0.499 | 0.89 |
| | | | 240/214 | Cancer | Control | Additive | 1.11 | 0.87, 1.42 | 0.411 | 0.89 |
| | | | 240/323 | Cancer | Control/Polyp | Dominant | 1.05 | 0.73, 1.52 | 0.780 | 0.95 |
| | | | 240/323 | Cancer | Control/Polyp | Recessive | 1.19 | 0.81, 1.74 | 0.381 | 0.89 |
| | | | 240/323 | Cancer | Control/Polyp | Additive | 1.08 | 0.86, 1.36 | 0.490 | 0.89 |
| | | | 355/323 | Adenoma/HGD/Cancer | Control/Polyp | Dominant | 1.00 | 0.71, 1.39 | 0.988 | 1.00 |
| | | | 355/323 | Adenoma/HGD/Cancer | Control/Polyp | Recessive | 1.15 | 0.81, 1.64 | 0.434 | 0.89 |
| | | | 355/323 | Adenoma/HGD/Cancer | Control/Polyp | Additive | 1.05 | 0.86, 1.29 | 0.650 | 0.94 |
| | | | 355/214 | Adenoma/HGD/Cancer | Control | Dominant | 1.14 | 0.80, 1.63 | 0.465 | 0.89 |
| | | | 355/214 | Adenoma/HGD/Cancer | Control | Recessive | 1.07 | 0.73, 1.57 | 0.715 | 0.94 |

| | | | | | | | | | | |
|---|---|---|---|---|---|---|---|---|---|---|
| | | | 355/214 | Adenoma/HGD/Cancer | Control | Additive | 1.08 | 0.86, 1.35 | 0.506 | 0.89 |
| | | | 96/214 | Adenoma/HGD | Control | Dominant | 0.96 | 0.57, 1.60 | 0.863 | 0.99 |
| | | | 96/214 | Adenoma/HGD | Control | Recessive | 1.04 | 0.59, 1.81 | 0.895 | 0.99 |
| | | | 96/214 | Adenoma/HGD | Control | Additive | 1.00 | 0.72, 1.37 | 0.975 | 1.00 |
| | | | 96/323 | Adenoma/HGD | Control/Polyp | Dominant | 0.87 | 0.54, 1.43 | 0.589 | 0.91 |
| | | | 96/323 | Adenoma/HGD | Control/Polyp | Recessive | 1.07 | 0.63, 1.81 | 0.815 | 0.97 |
| | | | 96/323 | Adenoma/HGD | Control/Polyp | Additive | 0.97 | 0.71, 1.32 | 0.840 | 0.99 |
| *TXNRD2* | rs9605031 | C>T | 208/266 | Polyp/Adenoma/HGD | Control | Dominant | 0.81 | 0.52, 1.25 | 0.339 | 0.89 |
| | | | 208/266 | Polyp/Adenoma/HGD | Control | Recessive | 1.42 | 0.91, 2.22 | 0.126 | 0.80 |
| | | | 208/266 | Polyp/Adenoma/HGD | Control | Additive | 1.05 | 0.80, 1.37 | 0.738 | 0.94 |
| | | | 234/266 | Cancer | Control | Dominant | 1.25 | 0.81, 1.97 | 0.323 | 0.89 |
| | | | 234/266 | Cancer | Control | Recessive | 1.09 | 0.69, 1.71 | 0.701 | 0.94 |
| | | | 234/266 | Cancer | Control | Additive | 1.13 | 0.85, 1.49 | 0.394 | 0.89 |
| | | | 256/476 | Cancer | Control/Polyp | Dominant | 1.15 | 0.84, 1.58 | 0.377 | 0.89 |
| | | | 256/476 | Cancer | Control/Polyp | Recessive | 1.08 | 0.55, 1.89 | 0.954 | 1.00 |
| | | | 256/476 | Cancer | Control/Polyp | Additive | 1.10 | 0.85, 1.41 | 0.466 | 0.89 |
| | | | 355/476 | Adenoma/HGD/Cancer | Control/Polyp | Dominant | 1.07 | 0.80, 1.42 | 0.656 | 0.94 |
| | | | 355/476 | Adenoma/HGD/Cancer | Control/Polyp | Recessive | 1.04 | 0.59, 1.81 | 0.900 | 0.99 |
| | | | 355/476 | Adenoma/HGD/Cancer | Control/Polyp | Additive | 1.05 | 0.84, 1.31 | 0.680 | 0.94 |
| | | | 355/266 | Adenoma/HGD/Cancer | Control | Dominant | 1.01 | 0.69, 1.48 | 0.975 | 1.00 |
| | | | 355/266 | Adenoma/HGD/Cancer | Control | Recessive | 1.24 | 0.84, 1.84 | 0.281 | 0.86 |
| | | | 355/266 | Adenoma/HGD/Cancer | Control | Additive | 1.09 | 0.86, 1.37 | 0.496 | 0.89 |
| | | | 97/266 | Adenoma/HGD | Control | Dominant | 0.77 | 0.45, 1.31 | 0.329 | 0.89 |
| | | | 97/266 | Adenoma/HGD | Control | Recessive | 1.53 | 0.89, 2.65 | 0.124 | 0.80 |
| | | | 97/266 | Adenoma/HGD | Control | Additive | 1.06 | 0.75, 1.48 | 0.745 | 0.94 |
| | | | 97/476 | Adenoma/HGD | Control/Polyp | Dominant | 0.88 | 0.56, 1.38 | 0.579 | 0.91 |
| | | | 97/476 | Adenoma/HGD | Control/Polyp | Recessive | 1.08 | 0.43, 0.47 | 0.860 | 0.99 |
| | | | 97/476 | Adenoma/HGD | Control/Polyp | Additive | 0.94 | 0.67, 1.34 | 0.710 | 0.94 |
| *BAX* | rs4645887 | T>A | 209/211 | Polyp/Adenoma/HGD | Control | Dominant | 1.17 | 1.81, 0.69 | 0.486 | 0.89 |
| | | | 209/211 | Polyp/Adenoma/HGD | Control | Recessive | 1.17 | 0.74, 1.84 | 0.505 | 0.89 |

| | | | | | | | | | | |
|---|---|---|---|---|---|---|---|---|---|---|
| | | | 209/211 | Polyp/Adenoma/HGD | Control | Additive | 1.12 | 0.86, 1.48 | 0.404 | 0.89 |
| | | | 235/211 | Cancer | Control | Dominant | 0.70 | 0.45, 1.09 | 0.441 | 0.89 |
| | | | 235/211 | Cancer | Control | Recessive | 0.77 | 0.49, 1.19 | 0.241 | 0.83 |
| | | | 235/211 | Cancer | Control | Additive | 0.80 | 0.61, 1.04 | 0.094 | 0.75 |
| | | | 235/319 | Cancer | Control/Polyp | Dominant | 0.89 | 0.63, 1.26 | 0.504 | 0.89 |
| | | | 235/319 | Cancer | Control/Polyp | Recessive | 0.84 | 0.52, 1.36 | 0.473 | 0.89 |
| | | | 235/319 | Cancer | Control/Polyp | Additive | 0.90 | 0.69, 1.15 | 0.398 | 0.89 |
| | | | 355/319 | Adenoma/HGD/Cancer | Control/Polyp | Dominant | 0.87 | 0.64, 1.19 | 0.389 | 0.89 |
| | | | 355/319 | Adenoma/HGD/Cancer | Control/Polyp | Recessive | 0.80 | 0.51, 1.25 | 0.927 | 0.99 |
| | | | 355/319 | Adenoma/HGD/Cancer | Control/Polyp | Additive | 0.88 | 0.69, 1.10 | 0.260 | 0.85 |
| | | | 355/211 | Adenoma/HGD/Cancer | Control | Dominant | 0.77 | 0.52, 1.14 | 0.193 | 0.82 |
| | | | 355/211 | Adenoma/HGD/Cancer | Control | Recessive | 0.81 | 0.55, 1.18 | 0.276 | 0.86 |
| | | | 355/211 | Adenoma/HGD/Cancer | Control | Additive | 0.12 | 0.67, 1.06 | 0.144 | 0.80 |
| | | | 98/211 | Adenoma/HGD | Control | Dominant | 1.03 | 0.60, 1.76 | 0.913 | 0.99 |
| | | | 98/211 | Adenoma/HGD | Control | Recessive | 1.39 | 0.81, 2.41 | 0.237 | 0.83 |
| | | | 98/211 | Adenoma/HGD | Control | Additive | 1.14 | 0.82, 1.59 | 0.440 | 0.89 |
| | | | 98/319 | Adenoma/HGD | Control/Polyp | Dominant | 0.83 | 0.52, 1.32 | 0.424 | 0.89 |
| | | | 98/319 | Adenoma/HGD | Control/Polyp | Recessive | 0.71 | 0.35, 1.43 | 0.342 | 0.89 |
| | | | 98/319 | Adenoma/HGD | Control/Polyp | Additive | 0.83 | 0.59, 1.17 | 0.280 | 0.86 |
| *FOXO3* | rs3813498 | C>T | 209/213 | Polyp/Adenoma/HGD | Control | Dominant | 0.85 | 0.58, 1.24 | 0.396 | 0.89 |
| | | | 209/213 | Polyp/Adenoma/HGD | Control | Recessive | 0.68 | 0.36, 1.25 | 0.218 | 0.83 |
| | | | 209/213 | Polyp/Adenoma/HGD | Control | Additive | 0.84 | 0.64, 1.12 | 0.233 | 0.83 |
| | | | 242/213 | Cancer | Control | Dominant | 1.10 | 0.76, 1.59 | 0.618 | 0.92 |
| | | | 242/213 | Cancer | Control | Recessive | 0.59 | 0.32, 1.08 | 0.087 | 0.75 |
| | | | 242/213 | Cancer | Control | Additive | 0.94 | 0.72, 1.24 | 0.677 | 0.94 |
| | | | 256/322 | Cancer | Control/Polyp | Dominant | 0.95 | 0.67, 1.36 | 0.784 | 0.95 |
| | | | 256/322 | Cancer | Control/Polyp | Recessive | 1.69 | 1.14, 3.01 | 0.276 | 0.86 |
| | | | 256/322 | Cancer | Control/Polyp | Additive | 1.02 | 0.75, 1.38 | 0.907 | 0.99 |
| | | | 338/322 | Adenoma/HGD/Cancer | Control/Polyp | Dominant | 0.69 | 0.52, 0.92 | **0.011** | 0.5 |
| | | | 338/322 | Adenoma/HGD/Cancer | Control/Polyp | Recessive | 1.70 | 0.70, 4.09 | 0.241 | 0.83 |

| | | | | | | | | | | |
|---|---|---|---|---|---|---|---|---|---|---|
| | | | 338/322 | Adenoma/HGD/Cancer | Control/Polyp | Additive | 1.07 | 0.81, 1.42 | 0.621 | 0.92 |
| | | | 338/213 | Adenoma/HGD/Cancer | Control | Dominant | 0.97 | 0.70, 1.35 | 0.872 | 0.99 |
| | | | 338/213 | Adenoma/HGD/Cancer | Control | Recessive | 0.63 | 0.37, 1.05 | 0.080 | 0.50 |
| | | | 338/213 | Adenoma/HGD/Cancer | Control | Additive | 0.89 | 0.69, 1.14 | 0.357 | 0.89 |
| | | | 96/213 | Adenoma/HGD | Control | Dominant | 0.91 | 0.56, 1.47 | 0.701 | 0.94 |
| | | | 96/213 | Adenoma/HGD | Control | Recessive | 0.68 | 0.31, 1.51 | 0.346 | 0.89 |
| | | | 96/213 | Adenoma/HGD | Control | Additive | 0.88 | 0.61, 1.25 | 0.471 | 0.89 |
| | | | 96/322 | Adenoma/HGD | Control/Polyp | Dominant | 1.21 | 0.24, 0.75 | 0.431 | 0.89 |
| | | | 96/322 | Adenoma/HGD | Control/Polyp | Recessive | 1.71 | 0.50, 5.79 | 0.315 | 0.89 |
| | | | 96/322 | Adenoma/HGD | Control/Polyp | Additive | 1.22 | 0.81, 1.84 | 0.336 | 0.89 |
| *FRZB* | rs17265803 | T>C | 209/215 | Polyp/Adenoma/HGD | Control | Dominant | 0.81 | 0.49, 1.33 | 0.401 | 0.89 |
| | | | 209/215 | Polyp/Adenoma/HGD | Control | Recessive | 0.00 | 0 | 0.999 | 1.00 |
| | | | 209/215 | Polyp/Adenoma/HGD | Control | Additive | 0.79 | 0.48, 1.24 | 0.346 | 0.89 |
| | | | 248/215 | Cancer | Control | Dominant | 1.13 | 0.72, 1.77 | 0.581 | 0.91 |
| | | | 248/215 | Cancer | Control | Recessive | 0.87 | 0.05, 13.94 | 0.919 | 0.99 |
| | | | 248/215 | Cancer | Control | Additive | 1.12 | 0.73, 1.73 | 0.604 | 0.91 |
| | | | 248/323 | Cancer | Control/Polyp | Dominant | 1.20 | 0.80, 1.81 | 0.370 | 0.89 |
| | | | 248/323 | Cancer | Control/Polyp | Recessive | 1.30 | 0.08, 20.95 | 0.852 | 0.99 |
| | | | 248/323 | Cancer | Control/Polyp | Additive | 1.20 | 0.81, 1.78 | 0.369 | 0.89 |
| | | | 355/323 | Adenoma/HGD/Cancer | Control/Polyp | Dominant | 1.10 | 0.75, 1.60 | 0.634 | 0.92 |
| | | | 355/323 | Adenoma/HGD/Cancer | Control/Polyp | Recessive | 0.94 | 0.06, 15.12 | 0.966 | 1.00 |
| | | | 355/323 | Adenoma/HGD/Cancer | Control/Polyp | Additive | 1.09 | 0.75, 1.58 | 0.645 | 0.94 |
| | | | 355/215 | Adenoma/HGD/Cancer | Control | Dominant | 0.98 | 0.65, 1.47 | 0.931 | 0.99 |
| | | | 355/215 | Adenoma/HGD/Cancer | Control | Recessive | 0.48 | 0.03, 7.64 | 0.600 | 0.91 |
| | | | 355/215 | Adenoma/HGD/Cancer | Control | Additive | 0.97 | 0.65, 1.42 | 0.876 | 0.99 |
| | | | 99/215 | Adenoma/HGD | Control | Dominant | 0.79 | 0.42, 1.48 | 0.457 | 0.89 |
| | | | 99/215 | Adenoma/HGD | Control | Recessive | 0.00 | 0 | 0.999 | 1.00 |
| | | | 99/215 | Adenoma/HGD | Control | Additive | 0.77 | 0.42, 1.43 | 0.414 | 0.89 |
| | | | 99/323 | Adenoma/HGD | Control/Polyp | Dominant | 0.84 | 0.46, 1.53 | 0.561 | 0.91 |
| | | | 99/323 | Adenoma/HGD | Control/Polyp | Recessive | 0.00 | 0 | 0.999 | 1.00 |

| | | | | | | | | |
|---|---|---|---|---|---|---|---|---|
| | 99/323 | Adenoma/HGD | Control/Polyp | Additive | 0.82 | 0.45, 1.49 | 0.524 | 0.89 |

Polyps were generally hyperplastic and less than 2mm, or 1 or 2 tubular adenomas <10mm.

Adenomas (advanced adenomas) were all >10mm, or at least 3 adenomas <10mm, and/or with villous or tubulovillous components.

Adenomas with high grade dysplasia (HGD) were advanced adenomas with at least 5% HGD.

SNP, Single Nucleotide Polymorphism; OR, Odd Ratio; CI, Confidence Interval; Ca/Co, Case/Control; HGD, High Grade Dysplasia.

*p-*value* obtained from logistic regression model (Additive, Dominant, Recessive) adjusted for age and sex; ***p*-value considered significant at < 0.05 and noted in bold.**

FDR- False Discovery Rate obtained by the Benjamini–Hochberg procedure.
