# Supplementary material for: The Associations of Selenoprotein Genetic Variants with the Risks of Colorectal Adenoma and Colorectal Cancer: Case–Control Studies in Irish and Czech Populations
Source: Nutrients. 2022 Jun 29;14(13):2718. doi: 10.3390/nu14132718 (PMC9268344; doi:10.3390/nu14132718)
Supplement: Supplementary file 1 [file nutrients-14-02718-s001.zip › S. Table S4 May 2022 DH.pdf]

Table S4: ORs, 95% CIs, p-values, and FDR corrections for the full stratified analyses of the Czech Republic cohort.

Gene	SNP	Allele	Subsite	Ca/Co	Test	OR	95%~~CI~~	P*	FDR
GPX1	rs9818758	G>A	Colon	487/638	Dominant	1.01	0.78, 1.31	0.929	0.99
			Colon	487/638	Recessive	0.85	0.42, 1.73	0.654	0.98
			Colon	487/638	Additive	0.99	0.79, 1.24	0.948	0.99
			Rectal	231/638	Dominant	1.01	0.73, 1.40	0.969	0.99
			Rectal	231/638	Recessive	0.70	0.26, 1.88	0.473	0.88
			Rectal	231/638	Additive	0.97	0.73, 1.30	0.845	0.99
GPX4	rs2074451	T>G	Colon	487/638	Dominant	0.91	0.70, 1.17	0.442	0.87
			Colon	487/638	Recessive	1.11	0.82, 1.52	0.494	0.88
			Colon	487/638	Additive	0.99	0.83, 1.17	0.891	0.99
			Rectal	231/638	Dominant	1.21	0.86, 1.70	0.274	0.87
			Rectal	231/638	Recessive	1.35	0.93, 1.97	0.115	0.87
			Rectal	231/638	Additive	1.20	0.96, 1.50	0.105	0.87
GPX5	rs445870	A>G	Colon	487/638	Dominant	0.91	0.71, 1.50	0.412	0.87
			Colon	487/638	Recessive	0.78	0.50, 1.21	0.266	0.87
			Colon	487/638	Additive	0.90	0.75, 1.08	0.266	0.87
			Rectal	231/638	Dominant	1.04	0.77, 1.41	0.818	0.99
			Rectal	231/638	Recessive	0.68	0.37, 1.25	0.218	0.87
			Rectal	231/638	Additive	0.96	0.76, 1.22	0.727	0.99
SELENOM	rs11705137	T>C	Colon	231/638	Dominant	1.00	0.77, 1.29	0.977	0.99
			Colon	487/638	Recessive	0.87	0.65, 1.17	0.366	0.87
			Colon	487/638	Additive	0.96	0.81, 1.13	0.592	0.98
			Rectal	231/638	Dominant	0.88	0.64, 1.22	0.443	0.87
			Rectal	231/638	Recessive	0.76	0.51, 1.12	0.158	0.87
			Rectal	231/638	Additive	0.87	0.70, 1.08	0.194	0.87
SELENON	rs11247710	G>C	Colon	487/638	Dominant	0.99	0.77, 1.27	0.922	0.99
			Colon	487/638	Recessive	0.97	0.70, 1.33	0.847	0.99
			Colon	487/638	Additive	0.99	0.83, 1.17	0.863	0.99
			Rectal	231/638	Dominant	1.16	1.61, 0.88	0.381	0.87
			Rectal	231/638	Recessive	0.91	0.60, 1.38	0.65	0.98
			Rectal	231/638	Additive	1.04	0.83, 1.30	0.722	0.99
SELENON	rs4659382	C>G	Colon	487/638	Dominant	0.91	0.71, 1.16	0.443	0.87
			Colon	487/638	Recessive	0.74	0.41, 1.32	0.308	0.87
			Colon	487/638	Additive	0.90	0.73, 1.11	0.313	0.87
			Rectal	231/638	Dominant	1.01	0.74, 1.38	0.949	0.99
			Rectal	231/638	Recessive	0.97	0.48, 1.95	0.923	0.99
			Rectal	231/638	Additive	1.00	0.77, 1.30	0.986	0.99
SELENOV	rs4802034	C>T	Colon	487/638	Dominant	0.97	0.76, 1.23	0.782	0.99
			Colon	487/638	Recessive	1.60	0.95, 2.70	0.075	0.87

			Colon	487/638	Additive	1.05	0.86, 1.28	0.652	0.98
			Rectal	231/638	Dominant	0.90	0.67, 1.23	0.517	0.89
			Rectal	231/638	Recessive	1.42	0.74, 2.75	0.296	0.87
			Rectal	231/638	Additive	0.98	0.76, 1.27	0.87	0.99
SEPSH1	rs2275129	C>G	Colon	487/638	Dominant	0.94	0.73, 1.21	0.627	0.98
			Colon	487/638	Recessive	1.11	0.82, 1.50	0.505	0.88
			Colon	487/638	Additive	1.00	0.85, 1.19	0.96	0.99
			Rectal	231/638	Dominant	1.03	0.74, 1.44	0.848	0.99
			Rectal	231/638	Recessive	1.46	1.01, 2.10	**0.044**	0.87
			Rectal	231/638	Additive	1.15	0.93, 1.43	0.202	0.87
TXNRD1	rs11111979	C>G	Colon	487/638	Dominant	1.01	0.77, 1.33	0.917	0.99
			Colon	487/638	Recessive	1.20	0.91, 1.58	0.209	0.87
			Colon	487/638	Additive	1.07	0.91, 1.26	0.415	0.87
			Rectal	231/638	Dominant	0.77	0.17, 0.55	0.122	0.87
			Rectal	231/638	Recessive	1.10	0.77, 1.58	0.605	0.98
			Rectal	231/638	Additive	0.93	0.75, 1.15	0.498	0.88
TXNRD1	rs7953266	C>T	Colon	487/638	Dominant	0.99	0.77, 1.27	0.949	0.99
			Colon	487/638	Recessive	1.17	0.86, 1.64	0.315	0.87
			Colon	487/638	Additive	1.05	0.88, 1.24	0.613	0.98
			Rectal	231/638	Dominant	0.79	0.58, 1.08	0.145	0.87
			Rectal	231/638	Recessive	1.08	0.72, 1.61	0.71	0.99
			Rectal	231/638	Additive	0.92	0.73, 1.14	0.425	0.87
BAX	rs4645887	T>A	Colon	487/638	Dominant	0.86	0.67, 1.10	0.228	0.87
			Colon	487/638	Recessive	1.16	0.84, 1.60	0.377	0.87
			Colon	487/638	Additive	0.97	0.82, 1.15	0.716	0.99
			Rectal	231/638	Dominant	0.93	0.68, 1.28	0.673	0.99
			Rectal	231/638	Recessive	1.01	0.67, 1.54	0.954	0.99
			Rectal	231/638	Additive	0.97	0.78, 1.21	0.791	0.99
FOXO3	rs3813498	C>T	Colon	487/638	Dominant	1.12	0.87, 1.43	0.39	0.87
			Colon	487/638	Recessive	1.08	0.60, 1.94	0.801	0.99
			Colon	487/638	Additive	1.09	0.88, 1.34	0.419	0.87
			Rectal	231/638	Dominant	0.82	0.60, 1.15	0.252	0.87
			Rectal	231/638	Recessive	0.75	0.32, 1.74	0.497	0.88
			Rectal	231/638	Additive	0.84	0.63, 1.12	0.231	0.87
FRZB	rs17265803	T>C	Colon	487/638	Dominant	1.17	0.89, 1.53	0.263	0.87
			Colon	487/638	Recessive	0.93	0.35, 2.45	0.875	0.99
			Colon	487/638	Additive	1.13	0.88, 1.45	0.327	0.87
			Rectal	231/638	Dominant	1.20	0.85, 1.70	0.297	0.87
			Rectal	231/638	Recessive	2.00	0.75, 5.31	0.166	0.87
			Rectal	231/638	Additive	1.23	0.15, 0.91	0.183	0.87

SNP, Single Nucleotide Polymorphism; OR, Odd Ratio; CI, Confidence Interval; Ca/Co, Case/Control.

The p-*value* obtained from logistic regression model (Additive, Dominant, Recessive), adjusted for age and sex; the *p*-value considered significant at < 0.05 and noted in bold.
FDR- False Discovery Rate obtained by the Benjamini–Hochberg procedure.
